# Supplementary material for: Effectiveness and safety of nintedanib in prevention of pulmonary fibrosis induced by bleomycin in malignant ovarian germ cell tumour: study protocol for a randomised, double-blind, placebo-controlled trial
Source: BMJ Open. 2023 Dec 9;13(12):e074963. doi: 10.1136/bmjopen-2023-074963 (PMC10729112; doi:10.1136/bmjopen-2023-074963)
Supplement: Supplementary data [file bmjopen-2023-074963supp001.pdf]

The standard normal deviate for $\alpha = Z_{\alpha} = 1.9600$

The standard normal deviate for $\beta = Z_{\beta} = 0.8416$

Pooled proportion = $P = (q_1 * P_1) + (q_0 * P_0) = 0.1450$

$A = Z_{\alpha}\sqrt{P(1-P)(1/q_1 + 1/q_0)} = 1.3802$

$B = Z_{\beta}\sqrt{P_1(1-P_1)(1/q_1) + P_0(1-P_0)(1/q_0)} = 0.5657$

$C = (P_1 - P_0)^2 = 0.0441$

Total group size = $N = (A+B)^2/C = 86$

Continuity correction (added to N for Group 0) = $CC = 1/(q_1 * |P_1 - P_0|) = 10$

Sample size (with continuity correction)

	N	Outcome+	Outcome-
Group 1:	53	2	51
Group 0:	53	13	40
Total:	106	15	91
