# Supplementary material for: Effectiveness and safety of nintedanib in prevention of pulmonary fibrosis induced by bleomycin in malignant ovarian germ cell tumour: study protocol for a randomised, double-blind, placebo-controlled trial
Source: BMJ Open. 2023 Dec 9;13(12):e074963. doi: 10.1136/bmjopen-2023-074963 (PMC10729112; doi:10.1136/bmjopen-2023-074963)
Supplement: Author's manuscript [file bmjopen-2023-074963.draft_revisions.pdf]

BMJ Open is committed to open peer review. As part of this commitment we make the peer review history of every article we publish publicly available.

When an article is published we post the peer reviewers' comments and the authors' responses online. We also post the versions of the paper that were used during peer review. These are the versions that the peer review comments apply to.

The versions of the paper that follow are the versions that were submitted during the peer review process. They are not the versions of record or the final published versions. They should not be cited or distributed as the published version of this manuscript.

BMJ Open is an open access journal and the full, final, typeset and author-corrected version of record of the manuscript is available on our site with no access controls, subscription charges or pay-per-view fees (http://bmjopen.bmj.com).

If you have any questions on BMJ Open's open peer review process please email info.bmjopen@bmj.com

BMJ Open

Effectiveness and Safety of Nintedanib in Prevention of Pulmonary Fibrosis Induced by Bleomycin in Malignant Ovarian Germ Cell Tumor: study protocol for a Randomized, double-blind, placebo-controlled Trial

Journal:	*BMJ Open*
Manuscript ID	bmjopen-2023-074963
Article Type:	Protocol
Date Submitted by the Author:	21-Apr-2023
Complete List of Authors:	Li, Sijian; Peking Union Medical College Hospital, Department of Obstetrics and Gynecology<br>Zhang, Xinyue; Peking Union Medical College Hospital, Department of Obstetrics and Gynecology<br>Yin, Min; Peking Union Medical College Hospital, Department of Obstetrics and Gynecology<br>Zhang, Tianyu; Peking Union Medical College Hospital, Department of Obstetrics and Gynecology<br>Zhang, Rundong; Peking Union Medical College Hospital, Department of Obstetrics and Gynecology<br>Yang, Jie; Peking Union Medical College Hospital, Department of Obstetrics and Gynecology<br>Yang, Jiaxin; Peking Union Medical College Hospital, Department of Obstetrics and Gynecology
Keywords:	Clinical Trial, Gynaecological oncology < GYNAECOLOGY, Protocols & guidelines < HEALTH SERVICES ADMINISTRATION & MANAGEMENT, ONCOLOGY

SCHOLARONE™
Manuscripts

Effectiveness and Safety of Nintedanib in Prevention of Pulmonary Fibrosis Induced by Bleomycin in Malignant Ovarian Germ Cell Tumor: study protocol for a Randomized, double-blind, placebo-controlled Trial

Sijian Li[1], Xinyue Zhang[1], Min Yin[1], Tianyu Zhang[1], Rundong Zhang[1], Jie Yang[1], Jiaxin Yang[1,*]

[1]National Clinical Research Center for Obstetric and Gynecologic Diseases, Department of Obstetrics and Gynecology, Peking Union Medical College Hospital, Chinese Academy of Medical Sciences, Peking Union Medical College, Beijing, People's Republic of China

***Correspondence**: Jiaxin Yang, MD, E-mail: yjxpumch@163.com

National Clinical Research Center for Obstetric and Gynecologic Diseases, Department of Obstetrics and Gynecology, Peking Union Medical College Hospital, Chinese Academy of Medical Sciences, Peking Union Medical College, Beijing, People's Republic of China

For peer review only

Abstract:

Introduction: Bleomycin is crucial and irreplaceable chemotherapy regimen for malignant ovarian germ cell tumors (MOGCTs) but its toxicities especially pulmonary fibrosis have limited the dose of treatment efficacy and decreased the patients' quality of life (QoL). Nintedanib has been approved for treating progressive fibrosing interstitial lung diseases and showed potential anti-tumor effects. This study aims to evaluate the effectiveness and safety of nintedanib in the prevention of pulmonary fibrosis induced by bleomycin in MOGCTs patients.

Methods and analysis: This is a multicenter, randomized, double-blinded, placebo-controlled clinical trial. We will enroll a total of 116 patients who will be randomly assigned to the nintedanib group and placebo group in a 1:1 ratio. Standard bleomycin, etoposide, and cisplatin (BEP) chemotherapy will be given to each MOGCT patient. Besides, patients assigned to nintedanib and the control group will be given oral nintedanib 150mg twice daily and placebo one tablet twice a day until one month after the last cycle of bleomycin therapy, respectively. The primary outcomes are a decline of pulmonary function (forced vital capacity, FVC; forced expiratory volume in 1 second, FEV1; FVC pred%; Carbon monoxide diffusion capacity, DLCO). The secondary outcomes are the QoL, oncological, and fertility outcomes of the patients. We will use electronic case report forms to record all the participants' data and SPSS V27.0/STATA V16.0/Graphpad Prism V8.0 to conduct statistical analysis.

Ethics and dissemination: The Ethics Committee of Peking Union Medical College Hospital has approved the study (I-23PJ400). Written informed consent will be obtained from all participants/guardians. Study results will be submitted to peer-reviewed medical journals for publication and presented at academic conferences.

Trial registration: The Chinese Clinical Trial Registry (ChiCTR) has approved the protocol (Register ID: ChiCTR2300070492; date of approval: April 13, 2023).

Strengths and limitations of this study

1. This trial aims to evaluate the effectiveness and the safety of nintedanib in MOGCTs patients receiving bleomycin chemotherapy.

2. This prospective, randomized, double-blinded, placebo-controlled design increases the reliability of the results and conclusions.

For peer review only

3. The noninvasive test of pulmonary function and other outcomes will enhance the feasibility and compliance of participants.

4. The limitation is that it may need more time to enroll enough participants due to the rarity and need a long-term follow-up to assess some secondary outcomes.

Introduction

Ovarian malignant germ cell tumors (MOGCTs) are heterogeneous non-epithelial ovarian malignant tumors that originate from the abnormal differentiation and malignant transformation of primordial germ cells of embryonic gonads. MOGCTs are rare and only account for about 3% of all ovarian malignancies, with an estimated incidence of 0.4/100,000 women years and mostly affected patients about 20 years [1, 2]. It is estimated that approximately 60-70% of MOGCTs patients are diagnosed with International Federation of Gynecology and Obstetrics (FIGO) stage I, and only less than 5% are with FIGO stage IV diseases upon initial treatment, therefore, the prognosis of MOGCTs patients is usually satisfactory [1, 3].

Currently, the standard treatment for MOGCTs patients can be a combination of surgery and adjuvant chemotherapy (bleomycin, etoposide, and cisplatin, BEP regimen) based on tumor stage and/or grade, while fertility-sparing surgery is always recommended in childbearing or young patients [4, 5]. Bleomycin is the crucial and irreplaceable regimen for chemotherapy in MOGCTs [6]. However, the toxicities associated with bleomycin including acute and later-onset pulmonary toxicity have limited the dose of the regimen, in terms of treatment efficacy, and have decreased the quality of life in these young patients [7]. The risk of pulmonary fibrosis is an important adverse effect of bleomycin, and it has been reported that pulmonary toxicity induced by bleomycin occurred in 6.8% to 46% of patients, with bleomycin-related mortality of about 3% to 5% [8]. Other adverse effects are less common, such as fever, hypersensitivity, and organizing pneumonitis [9].

Tumor relapse usually occurs within 2 years after treatment in MOGCTs patients [10]. Although chemotherapy after relapse can achieve a complete remission rate of about 75%, about one-third of advanced MOGCTs patients may experience recurrence or chemotherapy resistance in a short period, resulting in poor survival outcomes [7, 11]. Relapsed MOGCTs are usually not curable partly due to intolerable to bleomycin or do not have enough therapeutic doses of bleomycin, whereas other chemotherapy regimens are not effective [12].

For peer review only

Hence, it has always been emphasized to alleviate bleomycin-induced toxicities, especially pulmonary fibrosis. Although the exact mechanisms of pulmonary fibrosis caused by bleomycin remain uncertain, it has been proposed that inflammatory reaction and subsequent fibroplasia may play an important role in the pathophysiology of pulmonary fibrosis [13, 14]. Nintedanib is a small-molecule tyrosine kinase inhibitor that targets platelet-derived growth factor (PDGF) receptor-α and -β, fibroblast growth factor (FGF) receptor-1–3, and vascular endothelial growth factor (VEGF) receptor-1–3 [15]. Nintedanib has been approved for the treatment of idiopathic pulmonary fibrosis (IPF) in many countries, and several clinical trials have proven its effectiveness and safety in the treatment of IPF [16, 17]. Recently, Gundogan et al. also reported a 13-year-old lymphoma patient treated with a bleomycin-containing chemotherapy regimen who developed bleomycin-induced pneumonia that was successfully treated with nintedanib [18]. Moreover, nintedanib also showed anti-tumor effects in some reports [19, 20].

However, none of these studies provide evidence of nintedanib in the treatment of pulmonary fibrosis in MOGCTs. Therefore, a prospective, double-blinded, randomized control trial is designed to evaluate the effectiveness and safety of nintedanib in MOGCTs patients receiving bleomycin.

Objective

The primary aim of this randomized, control trial is to determine whether nintedanib can alleviate pulmonary function impairment (reduction decline of forced vital capacity [FVC], forced expiratory volume in 1 second [FEV1], FVC pred%, and Carbon monoxide diffusion capacity [DLCO]) in MOGCTs patients who receive BEP chemotherapy. The secondary objective is to evaluate the safety of nintedanib treatment, oncologic and fertility outcomes, and quality of life (QoL) in MOGCTs patients.

METHODS AND ANALYSIS

Study design

This is a prospective, randomized, double-blinded, placebo-controlled clinical trial that was registered at the Chinese Clinical Trial Registry on April 14, 2023 (ChiCTR2300070492). It has been approved by the Ethics Committee of Peking Union Medical College Hospital and will be

For peer review only

conducted in accordance with the Declaration of Helsinki and strictly follow the latest Consolidated Standards of Reporting Trials (CONSORT) recommendations [21], Standard Protocol Items: Recommendations

for Interventional Trials (SPIRIT) statement [22]. For all patients who will participate in this study, written informed consent will be obtained before treatment.

A total of 116 participants diagnosed with MOGCTs treated with BEP chemotherapy will be enrolled in the trial. The total screening and enrolling period will last for about 2.5 years in estimation, and they will be followed up for at least one year (up to 5 years) after treatment. The participants will be randomized and assigned to either the intervention group or the placebo group in a 1:1 ratio using a central randomization system. In addition to standard adjuvant chemotherapy, they will be given nintedanib (150mg) or placebo in granule form twice a day before chemotherapy till one year after the last cycle of chemotherapy.

Participants

The inclusion criteria are listed as follows:

(1) Newly diagnosed MOGCTs patients who will be treated with BEP chemotherapy or bleomycin-based chemotherapy;

(2) Age between 14 to 35 years;

(3) Pulmonary function test can be completed;

(4) FVC ≥ 45% of normal predicted value and DLCO ≥ 60% of normal predicted value;

(5) Patients who agree and sign the written informed consent, and with good compliance.

Patients who meet the following criteria will be excluded:

(1) Patients with pre-existing interstitial lung disease;

(2) Patients with severe medical diseases, severely impaired liver function (transaminase increased to 2.5 times the upper limit of normal), and severely impaired renal function (creatinine clearance rate <30mL/min);

(3) Combined with other reproductive system malignant tumors;

(4) Complicated with deep vein thrombosis, pulmonary embolism, myocardial infarction, cerebral infarction, and other diseases;

For peer review only

(5) Combined with other diseases requiring long-term hormone therapy;

(6) Patients who are considered not suitable to be enrolled by researchers.

Recruitment of patients and data collection

Participants inclusion

The recruit proclamation that includes a summarized description of the study will be released through webpages, in inpatient and outpatient areas. All the patients with MOGCTs (or their guardians in the case of juveniles) who visited our hospital will be evaluated whether they are suitable for entry into the clinical trial. The final pathology confirmation will be determined by two experienced pathologists at Peking Union Medical College Hospital. Then, for those potentially eligible MOGCTs patients, the study team will ask them or their guardians whether they are willing to participate in the study. Once written informed consent is obtained, members of the research team will collect the basic demographic information. Otherwise, the reasons why patients are excluded will also be recorded.

Study group assignment

After the exclusion and inclusion process, we will perform the concealed study group assignment based on the central randomization system. An independent statistician will generate a randomization sequence for all the eligible MOGCTs patients. The patients will be randomly allocated to either nintedanib (experimental) or placebo (control) groups. For all the MOGCT patients enrolled in this trial, a standard treatment, namely comprehensive fertility-sparing surgical staging, or radical surgical staging plus BEP chemotherapy for 3-4 cycles, in accordance with the ESMO guidelines [5], no matter whether they are assigned to which group. In addition to standard treatment, patients allocated to the nintedanib group will be given oral nintedanib 150 mg twice a day before the first cycle of BEP chemotherapy until one month after the last cycle of BEP chemotherapy. Accordingly, patients in the control group will be given an oral placebo one tablet per time, twice a day before the first cycle of BEP chemotherapy until one month after the last cycle of BEP chemotherapy. The nintedanib and placebo will be provided by the study team and the appearance will be the same, without identifiable labels or packaging. The patients will be requested to record the medication and return the packaging to researchers after treatment. The flow chart

For peer review only - http://bmjopen.bmj.com/site/about/guidelines.xhtml

(Figure 1) shows the simplified procedure of group allocation, interventions, and outcomes assessment.

Termination of therapy (nintedanib or placebo)

(1) The planned end of treatment (till one month after the last cycle of BEP chemotherapy);

(2) Termination of treatment due to severe adverse effects (AEs);

(3) Withdrawal of written informed consent.

Follow-up data collection

The disease-specific data, including tumor stage, pathological subtypes of MOGCTs, tumor markers' value, surgical options, and chemotherapy response, etc. will further be recorded in detail. The patients need to routinely follow up in outpatient before each chemotherapy cycle. The frequency of outpatient follow-up after finishing chemotherapy is set as follows: every three months within three years after treatment, and every 6 months between 3 and 5 years after treatment. Additional follow-up will be provided in case of special conditions, such as severe chemotherapy side effects, or tumor recurrence. A telephone follow-up will be a supplementary option for those patients who follow up in local hospitals or miss the appointment day.

During each follow-up, the patients will need to report the oncology outcomes, pulmonary function, QoL, menstrual conditions, and fertility outcomes for childbearing patients with the desire for fertility. The QoL assessment will be based on the general conditions and self-report questionaries listed as follow.

The privacy of patients will be rigorously protected throughout the whole study process. Any statistical analysis or publications of data can only be presented in an anonymization format.

Validated questionaries used in the trial

Quality of life will be mainly assessed by the Functional Assessment of Cancer Therapy Scale General (FACT-G) [23]. Other questionaries include Insomnia Severity Index (ISI) [24], the Physical Activity Questionnaire (IPAQ) [25], Peripheral Neurotoxicity Questionnaire (GOG-NTX) [26]. The FACT-G is a symptomatic scale including 12 items that can evaluate patients' symptoms, chemotherapy adverse effects, sexual and hormonal situations, etc. Similarly, ISI includes 7 items

For peer review only

to assess the quality of sleep and the severity of insomnia. IPAG shows validation in measuring overall physical activity and its frequency and intensity. GOG-NTX uses 11 items to describe peripheral nerve disorders, such as numbness, discomfort, arthralgia, deep sensitivity problems, and walking disorders. The detailed description has also been discussed in a previous study [27]. A combination of these questionaries can be helpful to demonstrate the life status of MOGCTs patients after treatment.

Outcomes

Primary outcomes

The primary outcomes of the study are the declined degree of pulmonary function in MOGCTs patients who receive BEP chemotherapy (decline of forced vital capacity [FVC], forced expiratory volume in 1 second [FEV1], FVC pred%, and Carbon monoxide diffusion capacity [DLCO]) revealed by respiratory function test. The secondary objective is to evaluate the safety of nintedanib treatment, oncologic and fertility outcomes, and quality of life (QoL) in MOGCTs patients.

The safety assessment of nintedanib is measured by patients' vital signs, auxiliary examinations, and AEs. The auxiliary examinations include blood tests (routine analysis, liver, and kidney function, etc.), urine, and stool examinations. We will record any AE during the treatment and follow-up period. The definition and classification of AEs based on the CTCAE5.0. Treatment will be continued in case of grade 1 or tolerable grade 2 AEs, and temporary termination of treatment will be applied in case of the first occurrence of intolerable grade 2 or grade 3 AEs until recovery to grade 0-1 or grade 2 AEs. However, permanent termination of nintedanib medication will be considered upon the second time of intolerable grade 2 or grade 3 AEs, no matter the previous toxicities or newly identified toxicities.

Sample size calculation

This study adopted a non-inferiority clinical trial design, and the sample size was calculated by using the sample size calculation website (https://sample-size.net). Based on the decline in lung function of patients treated with bleomycin at 1 year in the literature and the improvement in pulmonary function with nintedanib [28, 29], we set the decline of FVC and DLCO in the placebo group as over 8%. Based on the efficacy of nintedanib in the study of pulmonary fibrosis patients

For peer review only

[30], we set the decline of FVC and DLCO in the nintedanib group as 4%. Using a non-inferiority cut-off value of 10%, a one-sided α value of 0.025, and the test power of 0.09, we calculate the sample size of a single group as 53. Considering the group set a 1:1 ratio in two groups and 10% of the dropout cases during the trial, at least 116 patients will need to be included (Figure S1).

Data management and statistical analysis

We will use an electronic case report form (eCRF) to collect data, and an electronic database will be subsequently generated. All the investigators need to be authorized to access the data and will be required to maintain data confidentiality. A periodic indication to update outcomes' information during the follow-up will also be set up. After completion of the study, data will be stored for at least 20 years at a central data drive at the Peking Union Medical College Hospital. The datasets used and/or analyzed during the current study can be obtained from the principal investigator upon reasonable request. Moreover, a written signed data-sharing agreement will be required to get data access.

The variables will be described according to the distributions, namely, mean ± standard deviation (range) for normally distributed continuous variables, otherwise they are described as medians and interquartile ranges. Counts (percentages) will be used to present discrete variables. Methods for comparisons of variables between two groups will also be determined according to the data types. For oncology outcomes, including recurrence-free survival, overall survival, and disease-specific survival, we will use the Kaplan–Meier method (log-rank test). The univariate analysis will be performed to screen factors that should be subjected to multivariate analysis, and we set the cut-off *P*-values as 0.10 in univariate analysis. The Cox regression model or logistic regression model will be conducted in multivariate analysis. SPSS (version 20.0; SPSS Inc.), GraphPad Prism (version 8.0; GraphPad Software Inc.), or STATA (SE v12, StataCorp, College Station, TX, USA) software will be applied to perform the statistical analysis when appropriate. We set the statistical significance cut-off as 0.05 (two-tailed *P*-value).

ETHICS AND DISSEMINATION

The Ethics Committee of Peking Union Medical College Hospital has approved the study (I-

For peer review only

23PJ400). Written informed consent will be obtained from all participants/guardians. Study results will be submitted to peer-reviewed medical journals for publication and presented at academic conferences. The study will be conducted according to the principles of the Declaration of Helsinki.

CURRENT STATUS

Participants' enrollment will start on May 1st, 2023.

DISCUSSION

At present, bleomycin-induced pulmonary fibrosis remains a severe chemotherapy AE that significantly impairs the therapeutic efficacy and quality of life in MOGCTs patients. It was estimated that 8% of patients who received bleomycin may develop varied degrees of pulmonary fibrosis, and nearly 2% were lethal [29]. Although some optional chemotherapy regimens were proposed to decrease chemo toxicities in MOGCTs patients, bleomycin is a crucial and irreplaceable regimen for chemotherapy in MOGCTs patients [31, 32]. Nonetheless, the role of bleomycin is more important for recurrent MOGCTs due to dissatisfactory responses to other regimens [6, 12]. Therefore, how to alleviate pulmonary fibrosis during bleomycin-containing chemotherapy in MOGCTs has always raised great concerns.

The metabolism of bleomycin in the lung is poor because of the low activity of detoxifying enzymes and bleomycin hydrolase [8]. Hence, as the accumulation of bleomycin in the lung, it impairs the vascular system, resulting in the aggregation of inflammatory cells and fibroblasts into the lung tissue, which eventually leads to pulmonary fibrosis [33]. Nonetheless, the exact mechanisms of pulmonary fibrosis development remain unclear, and the time between bleomycin medication and pulmonary fibrosis varies greatly among different patients [34]. Routine prophylactic administration of anti-fibrosis drugs in patients receiving bleomycin may be practical due to unavailable for early identification of high-risk patients. In several clinical trials of nintedanib in treating progressive fibrosing interstitial lung diseases or idiopathic pulmonary fibrosis, nintedanib showed a significantly lower reduction in pulmonary function [18, 35]. Furthermore, nintedanib has been showing promising therapeutic efficacy in treating fibrosis caused by bleomycin-containing chemotherapy and potential additional benefits in combination with common chemotherapy regimens in cancer patients [18-20]. Besides, these studies showed relatively minor

For peer review only

AEs of nintedanib, revealing the safety of medication in targeted treating patients [35].

However, currently, there is limited evidence of nintedanib for the prevention or treatment of bleomycin-induced pulmonary fibrosis in MOGCT patients. There has been only limited low-grade evidence originating from case reports. Our prospective, double-blinded, randomized-controlled trial aims to provide high-quality on this issue, which will be helpful in the management of the severe AEs caused by bleomycin-containing chemotherapy in this population.

Acknowledgments

We want to thank all the patients enrolled in this study.

Authors' contributions

Jiaxin Yang is the principal investigator of this study who conceives the study design and modifies the manuscript. Sijian Li wrote the manuscript; Tianyu Zhang, Xinyue Zhang, Min Yin, and Rundong Zhang completed the establishment of the eCRF, participated in the literature review and trial registration; Jie Yang participated in the study design and designed the statistical analysis methods. Sijian Li, Tianyu Zhang, Xinyue Zhang, Min Yin, and Rundong Zhang will participate in patient recruitment, data collection, and statistical analysis. All authors read and approved the manuscript.

Funding

This study was supported by National High-Level Hospital Clinical Research Funding (2022-PUMCH-B-083) and Chinese Academy of Medical Sciences (CAMS) Innovation Fund for Medical Sciences (2022-I2M-C&T-B-023).

Disclaimer

The funding agency had no role in the design, and will not have any role during execution of the trial, data analyses, interpretation of the data, or decision to submit results.

Competing interest

The authors declare no conflict of interest.

For peer review only

Consent to participate and publication

Informed consent to participate and publication will be obtained for each enrolled patient.

Figure

Figure 1. The flow chart demonstrates the simplified process of group allocation, interventions, and outcomes assessment.

Supplementary files.

Figure S1. The sample size calculation process in this study.

References:

1. Park M, Lim J, Lee JA, Park HJ, Park BK, Lim MC, et al. Incidence and outcomes of malignant ovarian germ cell tumors in Korea, 1999-2017. Gynecol Oncol. 2021;163(1):79-84. doi: 10.1016/j.ygyno.2021.07.037 PMID: 34392955

2. Smith HO, Berwick M, Verschraegen CF, Wiggins C, Lansing L, Muller CY, et al. Incidence and survival rates for female malignant germ cell tumors. Obstet Gynecol. 2006;107(5):1075-1085. doi: 10.1097/01.AOG.0000216004.22588.ce PMID: 16648414

3. Torre LA, Trabert B, DeSantis CE, Miller KD, Samimi G, Runowicz CD, et al. Ovarian cancer statistics, 2018. CA Cancer J Clin. 2018;68(4):284-296. doi: 10.3322/caac.21456 PMID: 29809280

4. Euscher ED. Germ Cell Tumors of the Female Genital Tract. Surg Pathol Clin. 2019;12(2):621-649. doi: 10.1016/j.path.2019.01.005 PMID: 31097117

5. Ray-Coquard I, Morice P, Lorusso D, Prat J, Oaknin A, Pautier P, et al. Non-epithelial ovarian cancer: ESMO Clinical Practice Guidelines for diagnosis, treatment and follow-up. Ann Oncol. 2018;29(Suppl 4):iv1-iv18. doi: 10.1093/annonc/mdy001 PMID: 29697741

For peer review only

6. Bajorin DF, Bosl GJ. Bleomycin in germ cell tumor therapy: not all regimens are created equal. J Clin Oncol. 1997;15(5):1717-1719. doi: 10.1200/jco.1997.15.5.1717 PMID: 9164177

7. Simone CG, Markham MJ, Dizon DS. Chemotherapy in ovarian germ cell tumors: A systematic review. Gynecol Oncol. 2016;141(3):602-607. doi: 10.1016/j.ygyno.2016.02.007 PMID: 26873864

8. Fyfe AJ, McKay P. Toxicities associated with bleomycin. J R Coll Physicians Edinb. 2010;40(3):213-215. doi: 10.4997/jrcpe.2010.306 PMID: 21127762

9. Allawzi A, Elajaili H, Redente EF, Nozik-Grayck E. Oxidative Toxicology of Bleomycin: Role of the Extracellular Redox Environment. Curr Opin Toxicol. 2019;13:68-73. doi: 10.1016/j.cotox.2018.08.001 PMID: 31289762

10. Mangili G, Sigismondi C, Gadducci A, Cormio G, Scollo P, Tateo S, et al. Outcome and risk factors for recurrence in malignant ovarian germ cell tumors: a MITO-9 retrospective study. Int J Gynecol Cancer. 2011;21(8):1414-1421. doi: 10.1097/IGC.0b013e3182236582 PMID: 21795985

11. Reddy Ammakkanavar N, Matei D, Abonour R, Einhorn LH. High-dose chemotherapy for recurrent ovarian germ cell tumors. J Clin Oncol. 2015;33(2):226-227. doi: 10.1200/jco.2014.59.4325 PMID: 25452440

12. De Giorgi U, Casadei C, Bergamini A, Attademo L, Cormio G, Lorusso D, et al. Therapeutic Challenges for Cisplatin-Resistant Ovarian Germ Cell Tumors. Cancers (Basel). 2019;11(10). doi: 10.3390/cancers11101584 PMID: 31627378

13. Ortiz LA, Lasky J, Hamilton RF, Jr., Holian A, Hoyle GW, Banks W, et al. Expression of TNF and the necessity of TNF receptors in bleomycin-induced lung injury in mice. Exp Lung

For peer review only

Res. 1998;24(6):721-743. doi: 10.3109/01902149809099592 PMID: 9839161

14. Arif M, Basu A, Wolf KM, Park JK, Pommerolle L, Behee M, et al. An Integrative Multiomics Framework for Identification of Therapeutic Targets in Pulmonary Fibrosis. Adv Sci (Weinh). 2023:e2207454. doi: 10.1002/advs.202207454 PMID: 37038090

15. Hilberg F, Roth GJ, Krssak M, Kautschitsch S, Sommergruber W, Tontsch-Grunt U, et al. BIBF 1120: triple angiokinase inhibitor with sustained receptor blockade and good antitumor efficacy. Cancer Res. 2008;68(12):4774-4782. doi: 10.1158/0008-5472.Can-07-6307 PMID: 18559524

16. Flaherty KR, Wells AU, Cottin V, Devaraj A, Walsh SLF, Inoue Y, et al. Nintedanib in Progressive Fibrosing Interstitial Lung Diseases. N Engl J Med. 2019;381(18):1718-1727. doi: 10.1056/NEJMoa1908681 PMID: 31566307

17. Richeldi L, du Bois RM, Raghu G, Azuma A, Brown KK, Costabel U, et al. Efficacy and safety of nintedanib in idiopathic pulmonary fibrosis. N Engl J Med. 2014;370(22):2071-2082. doi: 10.1056/NEJMoa1402584 PMID: 24836310

18. Gundogan BD, Taskinlar S, Arikoglu T, Balci Y, Citak EC. Bleomycin-induced Pneumonitis in a Child Treated With Nintedanib: Report of the First Case in a Childhood. J Pediatr Hematol Oncol. 2022;44(2):e500-e502. doi: 10.1097/mph.0000000000002266 PMID: 35200223

19. Kai Y, Matsuda M, Fukuoka A, Hontsu S, Yamauchi M, Yoshikawa M, et al. Remarkable response of non-small cell lung cancer to nintedanib treatment in a patient with idiopathic pulmonary fibrosis. Thorac Cancer. 2021;12(9):1457-1460. doi: 10.1111/1759-7714.13935 PMID: 33742758

20. Kato R, Haratani K, Hayashi H, Sakai K, Sakai H, Kawakami H, et al. Nintedanib promotes

For peer review only

antitumour immunity and shows antitumour activity in combination with PD-1 blockade in mice: potential role of cancer-associated fibroblasts. Br J Cancer. 2021;124(5):914-924. doi: 10.1038/s41416-020-01201-z PMID: 33299131

21. Cheng CW, Wu TX, Shang HC, Li YP, Altman DG, Moher D, et al. CONSORT Extension for Chinese Herbal Medicine Formulas 2017: Recommendations, Explanation, and Elaboration. Ann Intern Med. 2017;167(2):112-121. doi: 10.7326/m16-2977 PMID: 28654980

22. Chan AW, Tetzlaff JM, Altman DG, Laupacis A, Gøtzsche PC, Krleža-Jerić K, et al. SPIRIT 2013 statement: defining standard protocol items for clinical trials. Ann Intern Med. 2013;158(3):200-207. doi: 10.7326/0003-4819-158-3-201302050-00583 PMID: 23295957

23. King MT, Stockler MR, Cella DF, Osoba D, Eton DT, Thompson J, et al. Meta-analysis provides evidence-based effect sizes for a cancer-specific quality-of-life questionnaire, the FACT-G. J Clin Epidemiol. 2010;63(3):270-281. doi: 10.1016/j.jclinepi.2009.05.001 PMID: 19716264

24. Morin CM, Belleville G, Bélanger L, Ivers H. The Insomnia Severity Index: psychometric indicators to detect insomnia cases and evaluate treatment response. Sleep. 2011;34(5):601-608. doi: 10.1093/sleep/34.5.601 PMID: 21532953

25. Keating XD, Zhou K, Liu X, Hodges M, Liu J, Guan J, et al. Reliability and Concurrent Validity of Global Physical Activity Questionnaire (GPAQ): A Systematic Review. Int J Environ Res Public Health. 2019;16(21). doi: 10.3390/ijerph16214128 PMID: 31717742

26. Cheng HL, Molassiotis A. Longitudinal validation and comparison of the Chinese version of the European Organization for Research and Treatment of Cancer Quality of Life-Chemotherapy-Induced Peripheral Neuropathy Questionnaire (EORTC QLQ-CIPN20) and the

For peer review only

403 Functional Assessment of Cancer-Gynecologic Oncology Group-Neurotoxicity subscale
404 (FACT/GOG-Ntx). Asia Pac J Clin Oncol. 2019;15(1):56-62. doi: 10.1111/ajco.13000 PMID:
405 29873180
406 27. Gernier F, Ahmed-Lecheheb D, Pautier P, Floquet A, Nadeau C, Frank S, et al. "Chronic
407 fatigue, quality of life and long-term side-effects of chemotherapy in patients treated for non-
408 epithelial ovarian cancer: national case-control protocol study of the GINECO-Vivrovaire rare
409 tumors INCa French network for rare malignant ovarian tumors". BMC Cancer.
410 2021;21(1):1147. doi: 10.1186/s12885-021-08864-8 PMID: 34702204
411 28. Wollin L, Distler JHW, Redente EF, Riches DWH, Stowasser S, Schlenker-Herceg R, et
412 al. Potential of nintedanib in treatment of progressive fibrosing interstitial lung diseases. Eur
413 Respir J. 2019;54(3). doi: 10.1183/13993003.00161-2019 PMID: 31285305
414 29. Delanoy N, Pécuchet N, Fabre E, Combe P, Juvin K, Pujade-Lauraine E, et al. Bleomycin-
415 Induced Pneumonitis in the Treatment of Ovarian Sex Cord-Stromal Tumors: A Systematic
416 Review and Meta-analysis. Int J Gynecol Cancer. 2015;25(9):1593-1598. doi:
417 10.1097/igc.0000000000000530 PMID: 26308607
418 30. Senoo S, Miyahara N, Taniguchi A, Oda N, Itano J, Higo H, et al. Nintedanib can be used
419 safely and effectively for idiopathic pulmonary fibrosis with predicted forced vital capacity ≤ 50%:
420 A multi-center retrospective analysis. PLoS One. 2020;15(8):e0236935. doi:
421 10.1371/journal.pone.0236935 PMID: 32853277
422 31. Chu R, Liu P, Chen J, Cheng X, Li K, Che Y, et al. Fertility and prognosis assessment
423 between bleomycin/etoposide/cisplatin and paclitaxel/carboplatin chemotherapy regimens in
424 the conservative treatment of malignant ovarian germ cell tumors: a multicenter and

retrospective study. J Gynecol Oncol. 2023;34(2):e12. doi: 10.3802/jgo.2023.34.e12 PMID: 36890292

32. Satoh T, Aoki Y, Kasamatsu T, Ochiai K, Takano M, Watanabe Y, et al. Administration of standard-dose BEP regimen (bleomycin+etoposide+cisplatin) is essential for treatment of ovarian yolk sac tumour. Eur J Cancer. 2015;51(3):340-351. doi: 10.1016/j.ejca.2014.12.004 PMID: 25559616

33. Huang TT, Hudson MM, Stokes DC, Krasin MJ, Spunt SL, Ness KK. Pulmonary outcomes in survivors of childhood cancer: a systematic review. Chest. 2011;140(4):881-901. doi: 10.1378/chest.10-2133 PMID: 21415131

34. Sleijfer S. Bleomycin-induced pneumonitis. Chest. 2001;120(2):617-624. doi: 10.1378/chest.120.2.617 PMID: 11502668

35. Bendstrup E, Wuyts W, Alfaro T, Chaudhuri N, Cornelissen R, Kreuter M, et al. Nintedanib in Idiopathic Pulmonary Fibrosis: Practical Management Recommendations for Potential Adverse Events. Respiration. 2019;97(2):173-184. doi: 10.1159/000495046 PMID: 30544129

For peer review only

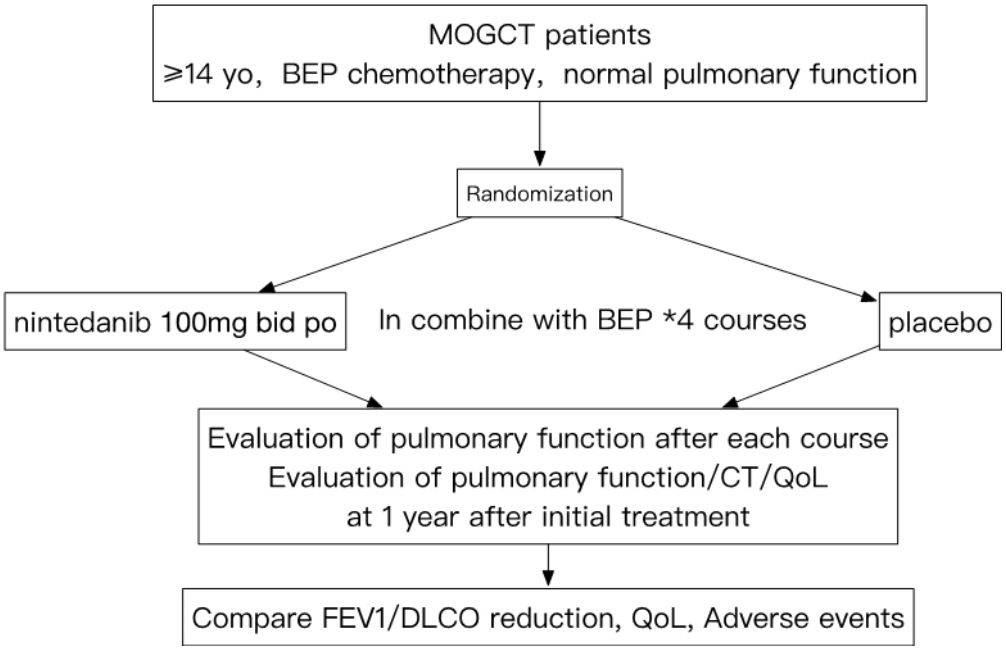

164x106mm (120 x 120 DPI)

The standard normal deviate for $\alpha = Z_\alpha = 1.9600$

The standard normal deviate for $\beta = Z_\beta = 0.8416$

Pooled proportion = $P = (q_1 * P_1) + (q_0 * P_0) = 0.1450$

$A = Z_\alpha \sqrt{P(1-P)(1/q_1 + 1/q_0)} = 1.3802$

$B = Z_\beta \sqrt{P_1(1-P_1)(1/q_1) + P_0(1-P_0)(1/q_0)} = 0.5657$

$C = (P_1 - P_0)^2 = 0.0441$

Total group size = $N = (A+B)^2/C = 86$

Continuity correction (added to N for Group 0) = $CC = 1/(q_1 * |P_1 - P_0|) = 10$

Sample size (with continuity correction)

	N	Outcome+	Outcome-
**Group 1:**	**53**	**2**	**51**
**Group 0:**	**53**	**13**	**40**
**Total:**	**106**	**15**	**91**

193x127mm (120 x 120 DPI)

BMJ Open

Effectiveness and Safety of Nintedanib in Prevention of Pulmonary Fibrosis Induced by Bleomycin in Malignant Ovarian Germ Cell Tumor: Study Protocol for a Randomized, Double-blind, Placebo-controlled Trial

Journal:	*BMJ Open*
Manuscript ID	bmjopen-2023-074963.R1
Article Type:	Protocol
Date Submitted by the Author:	27-Sep-2023

Complete List of Authors:	Li, Sijian; Peking Union Medical College Hospital, Department of Obstetrics and Gynecology<br>Zhang, Xinyue; Peking Union Medical College Hospital, Department of Obstetrics and Gynecology<br>Yin, Min; Peking Union Medical College Hospital, Department of Obstetrics and Gynecology<br>Zhang, Tianyu; Peking Union Medical College Hospital, Department of Obstetrics and Gynecology<br>Zhang, Rundong; Peking Union Medical College Hospital, Department of Obstetrics and Gynecology<br>Yang, Jie; Peking Union Medical College Hospital, Department of Obstetrics and Gynecology<br>Yang, Jiaxin; Peking Union Medical College Hospital, Department of Obstetrics and Gynecology

**Primary Subject Heading**:	Oncology
Secondary Subject Heading:	Obstetrics and gynaecology
Keywords:	Clinical Trial, Gynaecological oncology < GYNAECOLOGY, Protocols & guidelines < HEALTH SERVICES ADMINISTRATION & MANAGEMENT, ONCOLOGY

SCHOLARONE™
Manuscripts

Effectiveness and Safety of Nintedanib in Prevention of Pulmonary Fibrosis Induced by Bleomycin in Malignant Ovarian Germ Cell Tumor: Study Protocol for a Randomized, Double-blind, Placebo-controlled Trial

Sijian Li[1], Xinyue Zhang[1], Min Yin[1], Tianyu Zhang[1], Rundong Zhang[1], Jie Yang[1], Jiaxin Yang[1, *]

[1]National Clinical Research Center for Obstetric and Gynecologic Diseases, Department of Obstetrics and Gynecology, Peking Union Medical College Hospital, Chinese Academy of Medical Sciences, Peking Union Medical College, Beijing, People's Republic of China

***Correspondence**: Jiaxin Yang, MD, E-mail: yjxpumch@163.com

National Clinical Research Center for Obstetric and Gynecologic Diseases, Department of Obstetrics and Gynecology, Peking Union Medical College Hospital, Chinese Academy of Medical Sciences, Peking Union Medical College, Beijing, People's Republic of China

For peer review only

Abstract:

Introduction: Bleomycin is a crucial and irreplaceable chemotherapy regimen for malignant ovarian germ cell tumors (MOGCTs) but its toxicities especially pulmonary fibrosis have limited the dose of treatment efficacy and decreased the patients' quality of life (QoL). Nintedanib has been approved for treating progressive fibrosing interstitial lung diseases and has shown potential anti-tumor effects. This study aims to evaluate the effectiveness and safety of nintedanib in the prevention of pulmonary fibrosis induced by bleomycin in MOGCTs patients.

Methods and analysis: This is a multicenter, randomized, double-blinded, placebo-controlled clinical trial. We will enroll a total of 116 patients who will be randomly assigned to the nintedanib group and placebo group in a 1:1 ratio. Standard bleomycin, etoposide, and cisplatin (BEP) chemotherapy will be given to each MOGCT patient. Besides, patients assigned to nintedanib and the control group will be given oral nintedanib 150mg twice daily and placebo one tablet twice a day until one month after the last cycle of bleomycin therapy, respectively. The primary outcome is the decline of forced vital capacity (FVC). The secondary outcomes are the decline of other pulmonary function indices (forced expiratory volume in 1 second, FEV1; FVC pred%, Carbon monoxide diffusion capacity, DLCO), and the patients' QoL, oncological, and fertility outcomes. We will use electronic case report forms to record all the participants' data and SPSS V27.0/STATA V16.0/Graphpad Prism V8.0 to conduct statistical analysis.

Ethics and dissemination: The Ethics Committee of Peking Union Medical College Hospital has approved the study (I-23PJ400). Written informed consent will be obtained from all participants/guardians. Study results will be submitted to peer-reviewed medical journals for publication and presented at academic conferences.

Trial registration: The Chinese Clinical Trial Registry (ChiCTR) has approved the protocol (Register ID: ChiCTR2300070492; date of approval: April 13, 2023).

Strengths and limitations of this study

1. This trial aims to evaluate the effectiveness and the safety of nintedanib in MOGCTs patients receiving bleomycin chemotherapy.

2. This prospective, randomized, double-blinded, placebo-controlled design increases the reliability of the results and conclusions.

For peer review only

3. The noninvasive test of pulmonary function and other outcomes will enhance the feasibility and compliance of participants.

4. The limitation is that it may need more time to enroll enough participants due to the rarity and need a long-term follow-up to assess some secondary outcomes.

Introduction

Ovarian malignant germ cell tumors (MOGCTs) are heterogeneous non-epithelial ovarian malignant tumors that originate from the abnormal differentiation and malignant transformation of primordial germ cells of embryonic gonads. MOGCTs are rare and only account for about 3% of all ovarian malignancies, with an estimated incidence of 0.4/100,000 women years and mostly affected patients about 20 years [1, 2]. It is estimated that approximately 60-70% of MOGCTs patients are diagnosed with International Federation of Gynecology and Obstetrics (FIGO) stage I, and only less than 5% are with FIGO stage IV diseases upon initial treatment, therefore, the prognosis of MOGCTs patients is usually satisfactory [1, 3].

Currently, the standard treatment for MOGCTs patients can be a combination of surgery and adjuvant chemotherapy (bleomycin, etoposide, and cisplatin, BEP regimen) based on tumor stage and/or grade, while fertility-sparing surgery is always recommended in childbearing or young patients [4, 5]. Bleomycin is the crucial and irreplaceable regimen for chemotherapy in MOGCTs [6]. However, the toxicities associated with bleomycin including acute and later-onset pulmonary toxicity have limited the dose of the regimen, in terms of treatment efficacy, and have decreased the quality of life in these young patients [7]. The risk of pulmonary fibrosis is an important adverse effect of bleomycin, and it has been reported that pulmonary toxicity induced by bleomycin occurred in 6.8% to 46% of patients, with bleomycin-related mortality of about 3% to 5% [8]. Other adverse effects are less common, such as fever, hypersensitivity, and organizing pneumonitis [9].

Tumor relapse usually occurs within 2 years after treatment in MOGCTs patients [10]. Although chemotherapy after relapse can achieve a complete remission rate of about 75%, about one-third of advanced MOGCTs patients may experience recurrence or chemotherapy resistance in a short period, resulting in poor survival outcomes [7, 11]. Relapsed MOGCTs are usually not curable partly due to intolerance to bleomycin or not having enough therapeutic doses of bleomycin, whereas other chemotherapy regimens are not effective [12].

For peer review only

Hence, it has always been emphasized to alleviate bleomycin-induced toxicities, especially pulmonary fibrosis. Although the exact mechanisms of pulmonary fibrosis caused by bleomycin remain uncertain, it has been proposed that inflammatory reaction and subsequent fibroplasia may play an important role in the pathophysiology of pulmonary fibrosis [13, 14]. Nintedanib is a small-molecule tyrosine kinase inhibitor that targets platelet-derived growth factor (PDGF) receptor-α and -β, fibroblast growth factor (FGF) receptor-1–3, and vascular endothelial growth factor (VEGF) receptor-1–3 [15]. Nintedanib has been approved for the treatment of idiopathic pulmonary fibrosis (IPF) in many countries, and several clinical trials have proven its effectiveness and safety in the treatment of IPF [16, 17]. Recently, Gundogan et al. also reported a 13-year-old lymphoma patient treated with a bleomycin-containing chemotherapy regimen who developed bleomycin-induced pneumonia that was successfully treated with nintedanib [18]. Moreover, nintedanib also showed anti-tumor effects in some reports [19, 20].

However, none of these studies provide evidence of nintedanib in the treatment of pulmonary fibrosis in MOGCTs. Therefore, a prospective, double-blinded, randomized control trial is designed to evaluate the effectiveness and safety of nintedanib in MOGCTs patients receiving bleomycin.

Objective

The primary aim of this randomized, control trial is to determine whether nintedanib can alleviate the reduction decline of forced vital capacity (FVC) in MOGCTs patients who receive BEP chemotherapy. The secondary objective is to evaluate the decline of other pulmonary function indices (forced expiratory volume in 1 second, FEV1; FVC pred%, Carbon monoxide diffusion capacity, DLCO), the safety of nintedanib treatment, oncologic and fertility outcomes, and quality of life (QoL) in MOGCTs patients.

METHODS AND ANALYSIS

Study design

This is a prospective, randomized, double-blinded, placebo-controlled clinical trial that was registered at the Chinese Clinical Trial Registry on April 14, 2023 (ChiCTR2300070492). It has been approved by the Ethics Committee of Peking Union Medical College Hospital and will be

For peer review only - http://bmjopen.bmj.com/site/about/guidelines.xhtml

conducted in accordance with the Declaration of Helsinki and strictly follow the latest Consolidated Standards of Reporting Trials (CONSORT) recommendations [21], Standard Protocol Items: Recommendations for Interventional Trials (SPIRIT) statement [22] (Table S1). For all patients who will participate in this study, written informed consent will be obtained before treatment.

A total of 116 participants diagnosed with MOGCTs treated with BEP chemotherapy will be enrolled in the trial. The total screening and enrolling period will last for about 2.5 years in estimation, and they will be followed up for at least one year (up to 5 years) after treatment. The enrollment is planned to start on Oct. 1st, 2023 and end on Apr. 1st, 2026. The participants will be randomized and assigned to either the intervention group or the placebo group in a 1:1 ratio using a central randomization system. In addition to standard adjuvant chemotherapy, they will be given nintedanib (150mg) or placebo in granule form twice a day before chemotherapy till one month after the last cycle of chemotherapy.

Participants

The inclusion criteria are listed as follows:

(1) Newly diagnosed MOGCTs patients who will be treated with BEP chemotherapy or bleomycin-based chemotherapy;
(2) Age between 14 to 40 years;
(3) Pulmonary function test can be completed;
(4) FVC ≥ 45% of normal predicted value and DLCO ≥ 60% of normal predicted value;
(5) Patients who agree and sign the written informed consent, and with good compliance.

Patients who meet the following criteria will be excluded:

(1) Patients with pre-existing interstitial lung disease;
(2) Patients with severe medical diseases, severely impaired liver function (transaminase increased to 2.5 times the upper limit of normal), and severely impaired renal function (creatinine clearance rate <30mL/min);
(3) Combined with other reproductive system malignant tumors;
(4) Complicated with deep vein thrombosis, pulmonary embolism, myocardial infarction, cerebral infarction, and other diseases;

For peer review only

(5) Combined with other diseases requiring long-term hormone therapy;

(6) Patients with chronic obstructive pulmonary disease or pulmonary hypertension that influence pulmonary function;

(7) Patients who are considered not suitable to be enrolled by researchers.

Recruitment of patients and data collection

Participants inclusion

The recruit proclamation that includes a summarized description of the study will be released through webpages, in inpatient and outpatient areas. All the patients with MOGCTs (or their guardians in the case of juveniles) who visited our hospital will be evaluated whether they are suitable for entry into the clinical trial. The final pathology confirmation will be determined by two experienced pathologists at Peking Union Medical College Hospital. Then, for those potentially eligible MOGCTs patients, the study team will ask them or their guardians whether they are willing to participate in the study. Once written informed consent is obtained, members of the research team will collect the basic demographic information. Otherwise, the reasons why patients are excluded will also be recorded.

Study group assignment

After the exclusion and inclusion process, we will perform the concealed study group assignment based on the central randomization system. An independent statistician will generate a randomization sequence for all the eligible MOGCTs patients. The patients will be randomly allocated to either nintedanib (experimental) or placebo (control) groups. For all the MOGCT patients enrolled in this trial, a standard treatment, namely comprehensive fertility-sparing surgical staging, or radical surgical staging plus BEP chemotherapy for 3-4 cycles, in accordance with the ESMO guidelines [5], no matter whether they are assigned to which group. In addition to standard treatment, patients allocated to the nintedanib group will be given oral nintedanib 150 mg twice a day before the first cycle of BEP chemotherapy until one month after the last cycle of BEP chemotherapy. Accordingly, patients in the control group will be given an oral placebo one tablet per time, twice a day before the first cycle of BEP chemotherapy until one month after the last cycle of BEP chemotherapy. The nintedanib and placebo will be provided by the study team and the

For peer review only - http://bmjopen.bmj.com/site/about/guidelines.xhtml

appearance will be the same, without identifiable labels or packaging. The patients will be requested to record the medication and return the packaging to researchers after treatment. The flow chart (Figure 1) shows the simplified procedure of group allocation, interventions, and outcomes assessment.

Termination of therapy (nintedanib or placebo)

(1) The planned end of treatment (till one month after the last cycle of BEP chemotherapy);
(2) Termination of treatment due to severe adverse effects (AEs);
(3) Withdrawal of written informed consent.

Management in case of patients who could not complete pulmonary function test

In patients who may not be able to receive pulmonary function tests due to bleomycin-induced acute lung injury, arterial blood gases (ABGs) and high-resolution pulmonary CT will be performed to indirectly examine the pulmonary function. Meanwhile, it is also an adverse event that will be recorded in this trial. Pulmonary function tests will be performed after recovery or until those patients can complete this examination. For patients whose general condition has deteriorated due to MOGCT progression, if such patients had been enrolled in this trial, they would have recorded baseline and dynamic pulmonary function tests until they could not finish the pulmonary test. If they have not already finished the complete treatment for MOGCT and cannot continuously receive bleomycin-based chemotherapy, these adverse events will be recorded as positive events during the trial, and they will be dropped out from the trial. Additional enrollment of MOGCT patients will be an alternative option to solve this issue.

Follow-up data collection

The disease-specific data, including tumor stage, pathological subtypes of MOGCTs, tumor markers' value, surgical options, and chemotherapy response, etc. will further be recorded in detail. The patients need to routinely follow up in outpatient before each chemotherapy cycle. The frequency of outpatient follow-up after finishing chemotherapy is set as follows: every three months within three years after treatment, and every 6 months between 3 and 5 years after treatment. Additional follow-up will be provided in case of special conditions, such as severe chemotherapy

For peer review only

side effects, or tumor recurrence. A telephone follow-up will be a supplementary option for those patients who follow up in local hospitals or miss the appointment day. Importantly, the patients' smoking status, whether they receive oxygen therapy, and/or chest radiation before or during the treatment will be recorded in detail due to these factors may have a synergized effect with bleomycin.

During each follow-up, the patients will need to report the oncology outcomes, pulmonary function, QoL, menstrual conditions, and fertility outcomes for childbearing patients with the desire for fertility. The interval between bleomycin-containing chemotherapy and the onset of lung injury will be exactly recorded during the trial. The QoL assessment will be based on the general conditions and self-report questionaries listed as follows.

The privacy of patients will be rigorously protected throughout the whole study process. Any statistical analysis or publications of data can only be presented in an anonymization format.

Validated questionaries used in the trial

Quality of life will be mainly assessed by the Functional Assessment of Cancer Therapy Scale General (FACT-G) [23]. Other questionaries include the Insomnia Severity Index (ISI) [24], the Physical Activity Questionnaire (IPAQ) [25], Peripheral Neurotoxicity Questionnaire (GOG-NTX) [26]. The FACT-G is a symptomatic scale including 12 items that can evaluate patients' symptoms, chemotherapy adverse effects, sexual and hormonal situations, etc. Similarly, ISI includes 7 items to assess the quality of sleep and the severity of insomnia. IPAG shows validation in measuring overall physical activity and its frequency and intensity. GOG-NTX uses 11 items to describe peripheral nerve disorders, such as numbness, discomfort, arthralgia, deep sensitivity problems, and walking disorders. The detailed description has also been discussed in a previous study [27]. A combination of these questionaries can help demonstrate the life status of MOGCT patients after treatment.

Outcomes

Primary outcomes

The primary outcome of the study is the declined degree of FVC in MOGCT patients who receive BEP chemotherapy revealed by respiratory function test. The secondary outcomes are the decline of other pulmonary function indices (FEV1; FVC pred%, and DLCO), the safety of nintedanib

For peer review only

treatment, oncologic and fertility outcomes, and QoL in MOGCTs patients.

The safety assessment of nintedanib is measured by patients' vital signs, auxiliary examinations, and AEs. The auxiliary examinations include blood tests (routine analysis, liver, and kidney function, etc.), urine, and stool examinations. We will record any AE during the treatment and follow-up period. The definition and classification of AEs based on the CTCAE5.0. Treatment will be continued in case of grade 1 or tolerable grade 2 AEs, and temporary termination of treatment will be applied in case of the first occurrence of intolerable grade 2 or grade 3 AEs until recovery to grade 0-1 or grade 2 AEs. However, permanent termination of nintedanib medication will be considered upon the second time of intolerable grade 2 or grade 3 AEs, no matter the previous toxicities or newly identified toxicities.

Sample size calculation

This study adopted a non-inferiority clinical trial design, and the sample size was calculated by using the sample size calculation website (https://sample-size.net). Based on the decline in lung function of patients treated with bleomycin at 1 year in the literature and the improvement in pulmonary function with nintedanib [17, 28-30], we set the decline of FVC in the placebo group as over 8%. Based on the efficacy of nintedanib in the study of pulmonary fibrosis patients [16], we set the decline of FVC in the nintedanib group as 4%. Using a non-inferiority cut-off value of 10%, a one-sided α value of 0.025, and the test power of 0.09, we calculate the sample size of a single group as 53. Considering the group set a 1:1 ratio in two groups and 10% of the dropout cases during the trial, at least 116 patients will need to be included (Figure S1).

Patient and public involvement

We established a panel of 8 patients (in case of adult patients) or their guardians (children or adolescents' patients) was established for this trial. They helped to modify the study design and improve the final study protocol during online focus group meetings. Particularly, they proposed the recommendations on patient-related outcomes including the minimally important clinical differences, methods of recruitment and the design of data collection. They will be actively involved throughout all critical stages of the trial through regular parent panel meetings. The results of the

For peer review only - http://bmjopen.bmj.com/site/about/guidelines.xhtml

study will inform the participants or their guardians in case of children/adolescent's participants. Moreover, the additional burden of the intervention in this clinical trial will be fully covered by the hospital. However, the charge due to standard treatment of MOGCTs should be paid by the patients themselves.

Data management and statistical analysis

We will use an electronic case report form (eCRF) to collect data, and an electronic database will be subsequently generated. All the investigators need to be authorized to access the data and will be required to maintain data confidentiality. A periodic indication to update outcomes' information during the follow-up will also be set up. After completion of the study, data will be stored for at least 20 years at a central data drive at the Peking Union Medical College Hospital. The datasets used and/or analyzed during the current study can be obtained from the principal investigator upon reasonable request. Moreover, a written signed data-sharing agreement will be required to get data access.

The variables will be described according to the distributions, namely, mean ± standard deviation (range) for normally distributed continuous variables, otherwise they are described as medians and interquartile ranges. Counts (percentages) will be used to present discrete variables. Methods for comparisons of variables between two groups will also be determined according to the data types. For oncology outcomes, including recurrence-free survival, overall survival, and disease-specific survival, we will use the Kaplan–Meier method (log-rank test). The univariate analysis will be performed to screen factors that should be subjected to multivariate analysis, and we set the cut-off *P*-values as 0.10 in univariate analysis. The Cox regression model or logistic regression model will be conducted in multivariate analysis. SPSS (version 20.0; SPSS Inc., Chicago, IL), GraphPad Prism (version 8.0; GraphPad Software Inc., La Jolla, CA), or STATA (SE v12, StataCorp, College Station, TX, USA) software will be applied to perform the statistical analysis when appropriate. We set the statistical significance cut-off as 0.05 (two-tailed *P*-value).

ETHICS AND DISSEMINATION

The Ethics Committee of Peking Union Medical College Hospital has approved the study (I-23PJ400). Written informed consent will be obtained from all participants/guardians. Study results

For peer review only

will be submitted to peer-reviewed medical journals for publication and presented at academic conferences. The study will be conducted according to the principles of the Declaration of Helsinki.

CURRENT STATUS

Participants' enrollment is planned to start on Oct. 1st, 2023, and end on April 1st, 2026.

DISCUSSION

At present, bleomycin-induced pulmonary fibrosis remains a severe chemotherapy AE that significantly impairs the therapeutic efficacy and quality of life in MOGCT patients. It was estimated that 8% of patients who received bleomycin may develop varied degrees of pulmonary fibrosis, and nearly 2% were lethal [29]. Although some optional chemotherapy regimens were proposed to decrease chemo toxicities in MOGCT patients, bleomycin is a crucial and irreplaceable regimen for chemotherapy in MOGCTs patients [31, 32]. Nonetheless, the role of bleomycin is more important for recurrent MOGCTs due to dissatisfactory responses to other regimens [6, 12]. Therefore, how to alleviate pulmonary fibrosis during bleomycin-containing chemotherapy in MOGCTs has always raised great concerns.

The metabolism of bleomycin in the lung is poor because of the low activity of detoxifying enzymes and bleomycin hydrolase [8]. Hence, the accumulation of bleomycin in the lung, impairs the vascular system, resulting in the aggregation of inflammatory cells and fibroblasts into the lung tissue, which eventually leads to pulmonary fibrosis [33]. Nonetheless, the exact mechanisms of pulmonary fibrosis development remain unclear, and the time between bleomycin medication and pulmonary fibrosis varies greatly among different patients [34]. Routine prophylactic administration of anti-fibrosis drugs in patients receiving bleomycin may be practical due to the availability for early identification of high-risk patients. In several clinical trials of nintedanib in treating progressive fibrosing interstitial lung diseases or idiopathic pulmonary fibrosis, nintedanib showed a significantly lower reduction in pulmonary function [18, 35]. Furthermore, nintedanib has been showing promising therapeutic efficacy in treating fibrosis caused by bleomycin-containing chemotherapy and potential additional benefits in combination with common chemotherapy regimens in cancer patients [18-20]. Besides, these studies showed relatively minor AEs of nintedanib, revealing the safety of medication in targeted treating patients [35].

For peer review only - http://bmjopen.bmj.com/site/about/guidelines.xhtml

However, currently, there is limited evidence of nintedanib for the prevention or treatment of bleomycin-induced pulmonary fibrosis in MOGCT patients. There has been only limited low-grade evidence originating from case reports. Our prospective, double-blinded, randomized-controlled trial aims to provide high-quality on this issue, which will be helpful in the management of the severe AEs caused by bleomycin-containing chemotherapy in this population.

Acknowledgments

We want to thank patient advisers and all the patients enrolled in this study.

Authors' contributions

Jiaxin Yang is the principal investigator of this study who conceives the study design and modifies the manuscript. Sijian Li wrote the manuscript; Tianyu Zhang, Xinyue Zhang, Min Yin, and Rundong Zhang completed the establishment of the eCRF, participated in the literature review and trial registration; Jie Yang participated in the study design and designed the statistical analysis methods. Sijian Li, Tianyu Zhang, Xinyue Zhang, Min Yin, and Rundong Zhang will participate in patient recruitment, data collection, and statistical analysis. All authors read and approved the manuscript.

Funding

This study was supported by National High-Level Hospital Clinical Research Funding (2022-PUMCH-B-083) and Chinese Academy of Medical Sciences (CAMS) Innovation Fund for Medical Sciences (2022-I2M-C&T-B-023).

Disclaimer

The funding agency had no role in the design, and will not have any role during execution of the trial, data analyses, interpretation of the data, or decision to submit results.

Competing interest

The authors declare no conflict of interest.

For peer review only

Consent to participate and publication

Informed consent to participate and publication will be obtained for each enrolled patient.

Figure

Figure 1. The flow chart demonstrates the simplified process of group allocation, interventions, and outcomes assessment.

Supplementary files.

Table S1. The SPIRIT checklist in this study.

Figure S1. The sample size calculation process in this study.

References:

1. Park M, Lim J, Lee JA, Park HJ, Park BK, Lim MC, et al. Incidence and outcomes of malignant ovarian germ cell tumors in Korea, 1999-2017. Gynecol Oncol. 2021;163(1):79-84. doi: 10.1016/j.ygyno.2021.07.037 PMID: 34392955

2. Smith HO, Berwick M, Verschraegen CF, Wiggins C, Lansing L, Muller CY, et al. Incidence and survival rates for female malignant germ cell tumors. Obstet Gynecol. 2006;107(5):1075-1085. doi: 10.1097/01.AOG.0000216004.22588.ce PMID: 16648414

3. Torre LA, Trabert B, DeSantis CE, Miller KD, Samimi G, Runowicz CD, et al. Ovarian cancer statistics, 2018. CA Cancer J Clin. 2018;68(4):284-296. doi: 10.3322/caac.21456 PMID: 29809280

4. Euscher ED. Germ Cell Tumors of the Female Genital Tract. Surg Pathol Clin. 2019;12(2):621-649. doi: 10.1016/j.path.2019.01.005 PMID: 31097117

5. Ray-Coquard I, Morice P, Lorusso D, Prat J, Oaknin A, Pautier P, et al. Non-epithelial ovarian cancer: ESMO Clinical Practice Guidelines for diagnosis, treatment and follow-up. Ann Oncol. 2018;29(Suppl 4):iv1-iv18. doi: 10.1093/annonc/mdy001 PMID: 29697741

For peer review only

6. Bajorin DF, Bosl GJ. Bleomycin in germ cell tumor therapy: not all regimens are created equal. J Clin Oncol. 1997;15(5):1717-1719. doi: 10.1200/jco.1997.15.5.1717 PMID: 9164177

7. Simone CG, Markham MJ, Dizon DS. Chemotherapy in ovarian germ cell tumors: A systematic review. Gynecol Oncol. 2016;141(3):602-607. doi: 10.1016/j.ygyno.2016.02.007 PMID: 26873864

8. Fyfe AJ, McKay P. Toxicities associated with bleomycin. J R Coll Physicians Edinb. 2010;40(3):213-215. doi: 10.4997/jrcpe.2010.306 PMID: 21127762

9. Allawzi A, Elajaili H, Redente EF, Nozik-Grayck E. Oxidative Toxicology of Bleomycin: Role of the Extracellular Redox Environment. Curr Opin Toxicol. 2019;13:68-73. doi: 10.1016/j.cotox.2018.08.001 PMID: 31289762

10. Mangili G, Sigismondi C, Gadducci A, Cormio G, Scollo P, Tateo S, et al. Outcome and risk factors for recurrence in malignant ovarian germ cell tumors: a MITO-9 retrospective study. Int J Gynecol Cancer. 2011;21(8):1414-1421. doi: 10.1097/IGC.0b013e3182236582 PMID: 21795985

11. Reddy Ammakkanavar N, Matei D, Abonour R, Einhorn LH. High-dose chemotherapy for recurrent ovarian germ cell tumors. J Clin Oncol. 2015;33(2):226-227. doi: 10.1200/jco.2014.59.4325 PMID: 25452440

12. De Giorgi U, Casadei C, Bergamini A, Attademo L, Cormio G, Lorusso D, et al. Therapeutic Challenges for Cisplatin-Resistant Ovarian Germ Cell Tumors. Cancers (Basel). 2019;11(10). doi: 10.3390/cancers11101584 PMID: 31627378

13. Ortiz LA, Lasky J, Hamilton RF, Jr., Holian A, Hoyle GW, Banks W, et al. Expression of TNF and the necessity of TNF receptors in bleomycin-induced lung injury in mice. Exp Lung

For peer review only

Res. 1998;24(6):721-743. doi: 10.3109/01902149809099592 PMID: 9839161

14. Arif M, Basu A, Wolf KM, Park JK, Pommerolle L, Behee M, et al. An Integrative Multiomics Framework for Identification of Therapeutic Targets in Pulmonary Fibrosis. Adv Sci (Weinh). 2023:e2207454. doi: 10.1002/advs.202207454 PMID: 37038090

15. Hilberg F, Roth GJ, Krssak M, Kautschitsch S, Sommergruber W, Tontsch-Grunt U, et al. BIBF 1120: triple angiokinase inhibitor with sustained receptor blockade and good antitumor efficacy. Cancer Res. 2008;68(12):4774-4782. doi: 10.1158/0008-5472.Can-07-6307 PMID: 18559524

16. Flaherty KR, Wells AU, Cottin V, Devaraj A, Walsh SLF, Inoue Y, et al. Nintedanib in Progressive Fibrosing Interstitial Lung Diseases. N Engl J Med. 2019;381(18):1718-1727. doi: 10.1056/NEJMoa1908681 PMID: 31566307

17. Richeldi L, du Bois RM, Raghu G, Azuma A, Brown KK, Costabel U, et al. Efficacy and safety of nintedanib in idiopathic pulmonary fibrosis. N Engl J Med. 2014;370(22):2071-2082. doi: 10.1056/NEJMoa1402584 PMID: 24836310

18. Gundogan BD, Taskinlar S, Arikoglu T, Balci Y, Citak EC. Bleomycin-induced Pneumonitis in a Child Treated With Nintedanib: Report of the First Case in a Childhood. J Pediatr Hematol Oncol. 2022;44(2):e500-e502. doi: 10.1097/mph.0000000000002266 PMID: 35200223

19. Kai Y, Matsuda M, Fukuoka A, Hontsu S, Yamauchi M, Yoshikawa M, et al. Remarkable response of non-small cell lung cancer to nintedanib treatment in a patient with idiopathic pulmonary fibrosis. Thorac Cancer. 2021;12(9):1457-1460. doi: 10.1111/1759-7714.13935 PMID: 33742758

20. Kato R, Haratani K, Hayashi H, Sakai K, Sakai H, Kawakami H, et al. Nintedanib promotes

For peer review only

antitumour immunity and shows antitumour activity in combination with PD-1 blockade in mice: potential role of cancer-associated fibroblasts. Br J Cancer. 2021;124(5):914-924. doi: 10.1038/s41416-020-01201-z PMID: 33299131

21. Cheng CW, Wu TX, Shang HC, Li YP, Altman DG, Moher D, et al. CONSORT Extension for Chinese Herbal Medicine Formulas 2017: Recommendations, Explanation, and Elaboration. Ann Intern Med. 2017;167(2):112-121. doi: 10.7326/m16-2977 PMID: 28654980

22. Chan AW, Tetzlaff JM, Altman DG, Laupacis A, Gøtzsche PC, Krleža-Jerić K, et al. SPIRIT 2013 statement: defining standard protocol items for clinical trials. Ann Intern Med. 2013;158(3):200-207. doi: 10.7326/0003-4819-158-3-201302050-00583 PMID: 23295957

23. King MT, Stockler MR, Cella DF, Osoba D, Eton DT, Thompson J, et al. Meta-analysis provides evidence-based effect sizes for a cancer-specific quality-of-life questionnaire, the FACT-G. J Clin Epidemiol. 2010;63(3):270-281. doi: 10.1016/j.jclinepi.2009.05.001 PMID: 19716264

24. Morin CM, Belleville G, Bélanger L, Ivers H. The Insomnia Severity Index: psychometric indicators to detect insomnia cases and evaluate treatment response. Sleep. 2011;34(5):601-608. doi: 10.1093/sleep/34.5.601 PMID: 21532953

25. Keating XD, Zhou K, Liu X, Hodges M, Liu J, Guan J, et al. Reliability and Concurrent Validity of Global Physical Activity Questionnaire (GPAQ): A Systematic Review. Int J Environ Res Public Health. 2019;16(21). doi: 10.3390/ijerph16214128 PMID: 31717742

26. Cheng HL, Molassiotis A. Longitudinal validation and comparison of the Chinese version of the European Organization for Research and Treatment of Cancer Quality of Life-Chemotherapy-Induced Peripheral Neuropathy Questionnaire (EORTC QLQ-CIPN20) and the

For peer review only

Functional Assessment of Cancer-Gynecologic Oncology Group-Neurotoxicity subscale (FACT/GOG-Ntx). Asia Pac J Clin Oncol. 2019;15(1):56-62. doi: 10.1111/ajco.13000 PMID: 29873180

27. Gernier F, Ahmed-Lecheheb D, Pautier P, Floquet A, Nadeau C, Frank S, et al. "Chronic fatigue, quality of life and long-term side-effects of chemotherapy in patients treated for non-epithelial ovarian cancer: national case-control protocol study of the GINECO-Vivrovaire rare tumors INCa French network for rare malignant ovarian tumors". BMC Cancer. 2021;21(1):1147. doi: 10.1186/s12885-021-08864-8 PMID: 34702204

28. Wollin L, Distler JHW, Redente EF, Riches DWH, Stowasser S, Schlenker-Herceg R, et al. Potential of nintedanib in treatment of progressive fibrosing interstitial lung diseases. Eur Respir J. 2019;54(3). doi: 10.1183/13993003.00161-2019 PMID: 31285305

29. Delanoy N, Pécuchet N, Fabre E, Combe P, Juvin K, Pujade-Lauraine E, et al. Bleomycin-Induced Pneumonitis in the Treatment of Ovarian Sex Cord-Stromal Tumors: A Systematic Review and Meta-analysis. Int J Gynecol Cancer. 2015;25(9):1593-1598. doi: 10.1097/igc.0000000000000530 PMID: 26308607

30. Roncolato FT, Chatfield M, Houghton B, Toner G, Stockler M, Thomson D, et al. The effect of pulmonary function testing on bleomycin dosing in germ cell tumours. Intern Med J. 2016;46(8):893-898. doi: 10.1111/imj.13158 PMID: 27305276

31. Chu R, Liu P, Chen J, Cheng X, Li K, Che Y, et al. Fertility and prognosis assessment between bleomycin/etoposide/cisplatin and paclitaxel/carboplatin chemotherapy regimens in the conservative treatment of malignant ovarian germ cell tumors: a multicenter and retrospective study. J Gynecol Oncol. 2023;34(2):e12. doi: 10.3802/jgo.2023.34.e12 PMID:

For peer review only

36890292

32. Satoh T, Aoki Y, Kasamatsu T, Ochiai K, Takano M, Watanabe Y, et al. Administration of standard-dose BEP regimen (bleomycin+etoposide+cisplatin) is essential for treatment of ovarian yolk sac tumour. Eur J Cancer. 2015;51(3):340-351. doi: 10.1016/j.ejca.2014.12.004 PMID: 25559616

33. Huang TT, Hudson MM, Stokes DC, Krasin MJ, Spunt SL, Ness KK. Pulmonary outcomes in survivors of childhood cancer: a systematic review. Chest. 2011;140(4):881-901. doi: 10.1378/chest.10-2133 PMID: 21415131

34. Sleijfer S. Bleomycin-induced pneumonitis. Chest. 2001;120(2):617-624. doi: 10.1378/chest.120.2.617 PMID: 11502668

35. Bendstrup E, Wuyts W, Alfaro T, Chaudhuri N, Cornelissen R, Kreuter M, et al. Nintedanib in Idiopathic Pulmonary Fibrosis: Practical Management Recommendations for Potential Adverse Events. Respiration. 2019;97(2):173-184. doi: 10.1159/000495046 PMID: 30544129

For peer review only

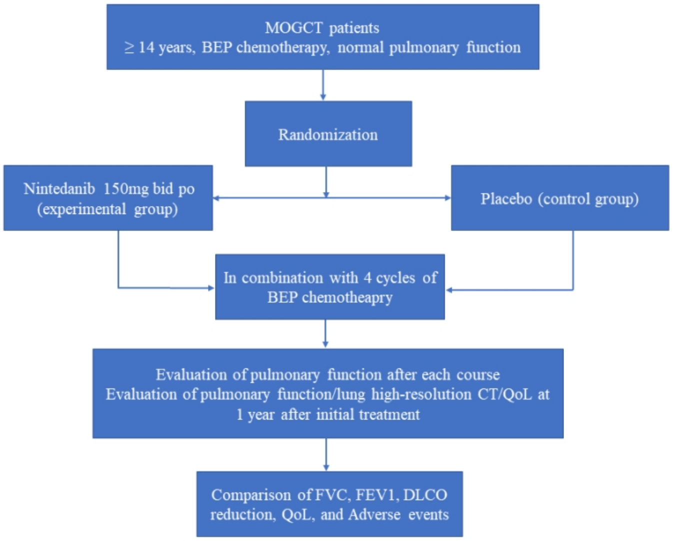

338x190mm (96 x 96 DPI)

The standard normal deviate for $\alpha = Z_\alpha = 1.9600$

The standard normal deviate for $\beta = Z_\beta = 0.8416$

Pooled proportion = $P = (q_1 {*} P_1) + (q_0 {*} P_0) = 0.1450$

$A = Z_\alpha \sqrt{P(1-P)(1/q_1 + 1/q_0)} = 1.3802$

$B = Z_\beta \sqrt{P_1(1-P_1)(1/q_1) + P_0(1-P_0)(1/q_0)} = 0.5657$

$C = (P_1 - P_0)^2 = 0.0441$

Total group size = $N = (A+B)^2/C = 86$

Continuity correction (added to N for Group 0) = $CC = 1/(q_1 * |P_1 - P_0|) = 10$

Sample size (with continuity correction)

	N	Outcome+	Outcome-
Group 1:	53	2	51
Group 0:	53	13	40
Total:	106	15	91

For peer review only

SPIRIT
STANDARD PROTOCOL ITEMS: RECOMMENDATIONS FOR INTERVENTIONAL TRIALS

SPIRIT 2013 Checklist: Recommended items to address in a clinical trial protocol and related documents*

Section/item	ItemNo	Description	Lines
**Administrative information**			
Title	1	Descriptive title identifying the study design, population, interventions, and, if applicable, trial acronym	1-3
Trial registration	2a	Trial identifier and registry name. If not yet registered, name of intended registry	36-37
	2b	All items from the World Health Organization Trial Registration Data Set	36-37
Protocol version	3	Date and version identifier	36-37
Funding	4	Sources and types of financial, material, and other support	319-321
Roles and responsibilities	5a	Names, affiliations, and roles of protocol contributors	323-325
	5b	Name and contact information for the trial sponsor	319-321
	5c	Role of study sponsor and funders, if any, in study design; collection, management, analysis, and interpretation of data; writing of the report; and the decision to submit the report for publication, including whether they will have ultimate authority over any of these activities	323-325
	5d	Composition, roles, and responsibilities of the coordinating centre, steering committee, endpoint adjudication committee, data management team, and other individuals or groups overseeing the trial, if applicable (see Item 21a for data monitoring committee)	323-325
**Introduction**			

For peer review only

For peer review only - http://bmjopen.bmj.com/site/about/guidelines.xhtml

Background and rationale	6a	Description of research question and justification for undertaking the trial, including summary of relevant studies (published and unpublished) examining benefits and harms for each intervention	50-88
	6b	Explanation for choice of comparators	50-88
Objectives	7	Specific objectives or hypotheses	91-96
Trial design	8	Description of trial design including type of trial (eg, parallel group, crossover, factorial, single group), allocation ratio, and framework (eg, superiority, equivalence, noninferiority, exploratory)	100-101
**Methods: Participants, interventions, and outcomes**			
Study setting	9	Description of study settings (eg, community clinic, academic hospital) and list of countries where data will be collected. Reference to where list of study sites can be obtained	101-103
Eligibility criteria	10	Inclusion and exclusion criteria for participants. If applicable, eligibility criteria for study centres and individuals who will perform the interventions (eg, surgeons, psychotherapists)	118-136
Interventions	11a	Interventions for each group with sufficient detail to allow replication, including how and when they will be administered	108-115; 154-163
	11b	Criteria for discontinuing or modifying allocated interventions for a given trial participant (eg, drug dose change in response to harms, participant request, or improving/worsening disease)	168-184
	11c	Strategies to improve adherence to intervention protocols, and any procedures for monitoring adherence (eg, drug tablet return, laboratory tests)	162-163
	11d	Relevant concomitant care and interventions that are permitted or prohibited during the trial	154-157
Outcomes	12	Primary, secondary, and other outcomes, including the specific measurement variable (eg, systolic blood pressure), analysis metric (eg, change from baseline, final value, time to event), method of aggregation (eg, median, proportion), and time point for each outcome. Explanation of the clinical relevance of chosen efficacy and harm outcomes is strongly recommended	220-232

For peer review only - http://bmjopen.bmj.com/site/about/guidelines.xhtml

Participant timeline	13	Time schedule of enrolment, interventions (including any run-ins and washouts), assessments, and visits for participants. A schematic diagram is highly recommended (see Figure)	186-202; 274
Sample size	14	Estimated number of participants needed to achieve study objectives and how it was determined, including clinical and statistical assumptions supporting any sample size calculations	235-243
Recruitment	15	Strategies for achieving adequate participant enrolment to reach target sample size	140-144
**Methods: Assignment of interventions (for controlled trials)**			
Allocation:			
Sequence generation	16a	Method of generating the allocation sequence (eg, computer-generated random numbers), and list of any factors for stratification. To reduce predictability of a random sequence, details of any planned restriction (eg, blocking) should be provided in a separate document that is unavailable to those who enrol participants or assign interventions	151-154
Allocation concealment mechanism	16b	Mechanism of implementing the allocation sequence (eg, central telephone; sequentially numbered, opaque, sealed envelopes), describing any steps to conceal the sequence until interventions are assigned	151-154
Implementation	16c	Who will generate the allocation sequence, who will enrol participants, and who will assign participants to interventions	152-153
Blinding (masking)	17a	Who will be blinded after assignment to interventions (eg, trial participants, care providers, outcome assessors, data analysts), and how	152-153
	17b	If blinded, circumstances under which unblinding is permissible, and procedure for revealing a participant's allocated intervention during the trial	151-154
**Methods: Data collection, management, and analysis**			
Data collection methods	18a	Plans for assessment and collection of outcome, baseline, and other trial data, including any related processes to promote data quality (eg, duplicate measurements, training of assessors) and a description of study instruments (eg, questionnaires, laboratory tests) along with their reliability and validity, if known. Reference to where data collection forms can be found, if not in the protocol	187-201

	18b	Plans to promote participant retention and complete follow-up, including list of any outcome data to be collected for participants who discontinue or deviate from intervention protocols	187-201
Data management	19	Plans for data entry, coding, security, and storage, including any related processes to promote data quality (eg, double data entry; range checks for data values). Reference to where details of data management procedures can be found, if not in the protocol	246-253
Statistical methods	20a	Statistical methods for analysing primary and secondary outcomes. Reference to where other details of the statistical analysis plan can be found, if not in the protocol	254-265
	20b	Methods for any additional analyses (eg, subgroup and adjusted analyses)	254-265
	20c	Definition of analysis population relating to protocol non-adherence (eg, as randomised analysis), and any statistical methods to handle missing data (eg, multiple imputation)	174-184
**Methods: Monitoring**			
Data monitoring	21a	Composition of data monitoring committee (DMC); summary of its role and reporting structure; statement of whether it is independent from the sponsor and competing interests; and reference to where further details about its charter can be found, if not in the protocol. Alternatively, an explanation of why a DMC is not needed	187-201
	21b	Description of any interim analyses and stopping guidelines, including who will have access to these interim results and make the final decision to terminate the trial	187-201
Harms	22	Plans for collecting, assessing, reporting, and managing solicited and spontaneously reported adverse events and other unintended effects of trial interventions or trial conduct	224-232
Auditing	23	Frequency and procedures for auditing trial conduct, if any, and whether the process will be independent from investigators and the sponsor	269-271
**Ethics and dissemination**			

Research ethics approval	24	Plans for seeking research ethics committee/institutional review board (REC/IRB) approval	32-33; 268-269
Protocol amendments	25	Plans for communicating important protocol modifications (eg, changes to eligibility criteria, outcomes, analyses) to relevant parties (eg, investigators, REC/IRBs, trial participants, trial registries, journals, regulators)	100-103
Consent or assent	26a	Who will obtain informed consent or assent from potential trial participants or authorised surrogates, and how (see Item 32)	269
	26b	Additional consent provisions for collection and use of participant data and biological specimens in ancillary studies, if applicable	269
Confidentiality	27	How personal information about potential and enrolled participants will be collected, shared, and maintained in order to protect confidentiality before, during, and after the trial	246-253
Declaration of interests	28	Financial and other competing interests for principal investigators for the overall trial and each study site	328
Access to data	29	Statement of who will have access to the final trial dataset, and disclosure of contractual agreements that limit such access for investigators	249-253
Ancillary and post-trial care	30	Provisions, if any, for ancillary and post-trial care, and for compensation to those who suffer harm from trial participation	268-271
Dissemination policy	31a	Plans for investigators and sponsor to communicate trial results to participants, healthcare professionals, the public, and other relevant groups (eg, via publication, reporting in results databases, or other data sharing arrangements), including any publication restrictions	268-271
	31b	Authorship eligibility guidelines and any intended use of professional writers	268-271
	31c	Plans, if any, for granting public access to the full protocol, participant-level dataset, and statistical code	252-253
**Appendices**			
Informed consent materials	32	Model consent form and other related documentation given to participants and authorised surrogates	331

For peer review only - http://bmjopen.bmj.com/site/about/guidelines.xhtml

Biological specimens	33	Plans for collection, laboratory evaluation, and storage of biological specimens for genetic or molecular analysis in the current trial and for future use in ancillary studies, if applicable	NA

*It is strongly recommended that this checklist be read in conjunction with the SPIRIT 2013 Explanation & Elaboration for important clarification on the items. Amendments to the protocol should be tracked and dated. The SPIRIT checklist is copyrighted by the SPIRIT Group under the Creative Commons "Attribution-NonCommercial-NoDerivs 3.0 Unported" license.

For peer review only

BMJ Open

Effectiveness and Safety of Nintedanib in Prevention of Pulmonary Fibrosis Induced by Bleomycin in Malignant Ovarian Germ Cell Tumor: Study Protocol for a Randomized, Double-blind, Placebo-controlled Trial

Journal:	*BMJ Open*
Manuscript ID	bmjopen-2023-074963.R2
Article Type:	Protocol
Date Submitted by the Author:	29-Nov-2023

Complete List of Authors:	Li, Sijian; Peking Union Medical College Hospital, Department of Obstetrics and Gynecology<br>Zhang, Xinyue; Peking Union Medical College Hospital, Department of Obstetrics and Gynecology<br>Yin, Min; Peking Union Medical College Hospital, Department of Obstetrics and Gynecology<br>Zhang, Tianyu; Peking Union Medical College Hospital, Department of Obstetrics and Gynecology<br>Zhang, Rundong; Peking Union Medical College Hospital, Department of Obstetrics and Gynecology<br>Yang, Jie; Peking Union Medical College Hospital, Department of Obstetrics and Gynecology<br>Yang, Jiaxin; Peking Union Medical College Hospital, Department of Obstetrics and Gynecology

**Primary Subject Heading**:	Oncology
Secondary Subject Heading:	Obstetrics and gynaecology
Keywords:	Clinical Trial, Gynaecological oncology < GYNAECOLOGY, Protocols & guidelines < HEALTH SERVICES ADMINISTRATION & MANAGEMENT, ONCOLOGY

SCHOLARONE™ Manuscripts

Effectiveness and Safety of Nintedanib in Prevention of Pulmonary Fibrosis Induced by Bleomycin in Malignant Ovarian Germ Cell Tumor: Study Protocol for a Randomized, Double-blind, Placebo-controlled Trial

Sijian Li[1], Xinyue Zhang[1], Min Yin[1], Tianyu Zhang[1], Rundong Zhang[1], Jie Yang[1], Jiaxin Yang[1,*]

[1]National Clinical Research Center for Obstetric and Gynecologic Diseases, Department of Obstetrics and Gynecology, Peking Union Medical College Hospital, Chinese Academy of Medical Sciences, Peking Union Medical College, Beijing, People's Republic of China

***Correspondence**: Jiaxin Yang, MD, E-mail: yjxpumch@163.com

National Clinical Research Center for Obstetric and Gynecologic Diseases, Department of Obstetrics and Gynecology, Peking Union Medical College Hospital, Chinese Academy of Medical Sciences, Peking Union Medical College, Beijing, People's Republic of China

For peer review only

Abstract:

Introduction: Bleomycin is a crucial and irreplaceable chemotherapy regimen for malignant ovarian germ cell tumors (MOGCTs) but its toxicities especially pulmonary fibrosis have limited the dose of treatment efficacy and decreased the patients' quality of life (QoL). Nintedanib has been approved for treating progressive fibrosing interstitial lung diseases and has shown potential anti-tumor effects. This study aims to evaluate the effectiveness and safety of nintedanib in the prevention of pulmonary fibrosis induced by bleomycin in MOGCTs patients.

Methods and analysis: This is a multicenter, randomized, double-blinded, placebo-controlled clinical trial. We will enroll a total of 128 patients who will be randomly assigned to the nintedanib group and placebo group in a 1:1 ratio. Standard bleomycin, etoposide, and cisplatin (BEP) chemotherapy will be given to each MOGCT patient. Besides, patients assigned to nintedanib and the control group will be given oral nintedanib 150mg twice daily and placebo one tablet twice a day until one month after the last cycle of bleomycin therapy, respectively. The primary outcome is the decline of forced vital capacity (FVC). The secondary outcomes are the decline of other pulmonary function indices (forced expiratory volume in 1 second, FEV1; FVC pred%, Carbon monoxide diffusion capacity, DLCO), and the patients' QoL, oncological, and fertility outcomes. We will use electronic case report forms to record all the participants' data and SPSS V27.0/STATA V16.0/Graphpad Prism V8.0 to conduct statistical analysis.

Ethics and dissemination: The Ethics Committee of Peking Union Medical College Hospital has approved the study (I-23PJ400). Written informed consent will be obtained from all participants/guardians. Study results will be submitted to peer-reviewed medical journals for publication and presented at academic conferences.

Trial registration: The Chinese Clinical Trial Registry (ChiCTR) has approved the protocol (Register ID: ChiCTR2300070492; date of approval: April 13, 2023).

Strengths and limitations of this study

1. This trial aims to evaluate the effectiveness and the safety of nintedanib in MOGCTs patients receiving bleomycin chemotherapy.

2. This prospective, randomized, double-blinded, placebo-controlled design increases the reliability of the results and conclusions.

For peer review only

3. The noninvasive test of pulmonary function and other outcomes will enhance the feasibility and compliance of participants.

4. The limitation is that it may need more time to enroll enough participants due to the rarity and need a long-term follow-up to assess some secondary outcomes and the single-center nature.

Introduction

Ovarian malignant germ cell tumors (MOGCTs) are heterogeneous non-epithelial ovarian malignant tumors that originate from the abnormal differentiation and malignant transformation of primordial germ cells of embryonic gonads. MOGCTs are rare and only account for about 3% of all ovarian malignancies, with an estimated incidence of 0.4/100,000 women years and mostly affected patients about 20 years [1, 2]. It is estimated that approximately 60-70% of MOGCTs patients are diagnosed with International Federation of Gynecology and Obstetrics (FIGO) stage I, and only less than 5% are with FIGO stage IV diseases upon initial treatment, therefore, the prognosis of MOGCTs patients is usually satisfactory [1, 3].

Currently, the standard treatment for MOGCTs patients can be a combination of surgery and adjuvant chemotherapy (bleomycin, etoposide, and cisplatin, BEP regimen) based on tumor stage and/or grade, while fertility-sparing surgery is always recommended in childbearing or young patients [4, 5]. Bleomycin is the crucial and irreplaceable regimen for chemotherapy in MOGCTs [6]. However, the toxicities associated with bleomycin including acute and later-onset pulmonary toxicity have limited the dose of the regimen, in terms of treatment efficacy, and have decreased the quality of life in these young patients [7]. The risk of pulmonary fibrosis is an important adverse effect of bleomycin, and it has been reported that pulmonary toxicity induced by bleomycin occurred in 6.8% to 46% of patients, with bleomycin-related mortality of about 3% to 5% [8]. Other adverse effects are less common, such as fever, hypersensitivity, and organizing pneumonitis [9].

Tumor relapse usually occurs within 2 years after treatment in MOGCTs patients [10]. Although chemotherapy after relapse can achieve a complete remission rate of about 75%, about one-third of advanced MOGCTs patients may experience recurrence or chemotherapy resistance in a short period, resulting in poor survival outcomes [7, 11]. Relapsed MOGCTs are usually not curable partly due to intolerance to bleomycin or not having enough therapeutic doses of bleomycin, whereas other chemotherapy regimens are not effective [12].

For peer review only

Hence, it has always been emphasized to alleviate bleomycin-induced toxicities, especially pulmonary fibrosis. Although the exact mechanisms of pulmonary fibrosis caused by bleomycin remain uncertain, it has been proposed that inflammatory reaction and subsequent fibroplasia may play an important role in the pathophysiology of pulmonary fibrosis [13, 14]. Nintedanib is a small-molecule tyrosine kinase inhibitor that targets platelet-derived growth factor (PDGF) receptor-α and -β, fibroblast growth factor (FGF) receptor-1–3, and vascular endothelial growth factor (VEGF) receptor-1–3 [15]. Nintedanib has been approved for the treatment of idiopathic pulmonary fibrosis (IPF) in many countries, and several clinical trials have proven its effectiveness and safety in the treatment of IPF [16, 17]. Recently, Gundogan et al. also reported a 13-year-old lymphoma patient treated with a bleomycin-containing chemotherapy regimen who developed bleomycin-induced pneumonia that was successfully treated with nintedanib [18]. Moreover, nintedanib also showed anti-tumor effects in some reports [19, 20].

However, none of these studies provide evidence of nintedanib in the treatment of pulmonary fibrosis in MOGCTs. Therefore, a prospective, double-blinded, randomized control trial is designed to evaluate the effectiveness and safety of nintedanib in MOGCTs patients receiving bleomycin.

Objective

The primary aim of this randomized, control trial is to determine whether nintedanib can alleviate the reduction decline of forced vital capacity (FVC) in MOGCTs patients who receive BEP chemotherapy. The secondary objective is to evaluate the decline of other pulmonary function indices (forced expiratory volume in 1 second, FEV1; FVC pred%, Carbon monoxide diffusion capacity, DLCO), the safety of nintedanib treatment, oncologic and fertility outcomes, and quality of life (QoL) in MOGCTs patients.

METHODS AND ANALYSIS

Study design

This is a prospective, randomized, double-blinded, placebo-controlled clinical trial that was registered at the Chinese Clinical Trial Registry on April 14, 2023 (ChiCTR2300070492). It has been approved by the Ethics Committee of Peking Union Medical College Hospital and will be

conducted in accordance with the Declaration of Helsinki and strictly follow the latest Consolidated Standards of Reporting Trials (CONSORT) recommendations [21], Standard Protocol Items: Recommendations for Interventional Trials (SPIRIT) statement [22] (Table S1). For all patients who will participate in this study, written informed consent will be obtained before treatment.

A total of 116 participants diagnosed with MOGCTs treated with BEP chemotherapy will be enrolled in the trial. The total screening and enrolling period will last for about 2.5 years in estimation, and they will be followed up for at least one year (up to 5 years) after treatment. The enrollment is planned to start on Oct. 1st, 2023 and end on Apr. 1st, 2026. The participants will be randomized and assigned to either the intervention group or the placebo group in a 1:1 ratio using a central randomization system. In addition to standard adjuvant chemotherapy, they will be given nintedanib (150mg) or placebo in granule form twice a day before chemotherapy till one month after the last cycle of chemotherapy.

Participants

The inclusion criteria are listed as follows:

(1) Newly diagnosed MOGCTs patients who will be treated with BEP chemotherapy or bleomycin-based chemotherapy;

(2) Age between 14 to 40 years;

(3) Pulmonary function test can be completed;

(4) FVC ≥ 45% of normal predicted value and DLCO ≥ 60% of normal predicted value;

(5) Patients who agree and sign the written informed consent, and with good compliance.

Patients who meet the following criteria will be excluded:

(1) Patients with pre-existing interstitial lung disease;

(2) Patients with severe medical diseases, severely impaired liver function (transaminase increased to 2.5 times the upper limit of normal), and severely impaired renal function (creatinine clearance rate <30mL/min);

(3) Combined with other reproductive system malignant tumors;

(4) Complicated with deep vein thrombosis, pulmonary embolism, myocardial infarction, cerebral infarction, and other diseases;

For peer review only

(5) Combined with other diseases requiring long-term hormone therapy;

(6) Patients with chronic obstructive pulmonary disease or pulmonary hypertension that influence pulmonary function;

(7) Patients who are considered not suitable to be enrolled by researchers.

Recruitment of patients and data collection

Participants inclusion

The recruit proclamation that includes a summarized description of the study will be released through webpages, in inpatient and outpatient areas. All the patients with MOGCTs (or their guardians in the case of juveniles) who visited our hospital will be evaluated whether they are suitable for entry into the clinical trial. The final pathology confirmation will be determined by two experienced pathologists at Peking Union Medical College Hospital. Then, for those potentially eligible MOGCTs patients, the study team will ask them or their guardians whether they are willing to participate in the study. Once written informed consent is obtained, members of the research team will collect the basic demographic information. Otherwise, the reasons why patients are excluded will also be recorded.

Study group assignment

After the exclusion and inclusion process, we will perform the concealed study group assignment based on the central randomization system. An independent statistician will generate a randomization sequence for all the eligible MOGCTs patients. The patients will be randomly allocated to either nintedanib (experimental) or placebo (control) groups. For all the MOGCT patients enrolled in this trial, a standard treatment, namely comprehensive fertility-sparing surgical staging, or radical surgical staging plus BEP chemotherapy for 3-4 cycles, in accordance with the ESMO guidelines [5], no matter whether they are assigned to which group. In addition to standard treatment, patients allocated to the nintedanib group will be given oral nintedanib 150 mg twice a day before the first cycle of BEP chemotherapy until one month after the last cycle of BEP chemotherapy. Accordingly, patients in the control group will be given an oral placebo one tablet per time, twice a day before the first cycle of BEP chemotherapy until one month after the last cycle of BEP chemotherapy. The nintedanib and placebo will be provided by the study team and the

For peer review only - http://bmjopen.bmj.com/site/about/guidelines.xhtml

appearance will be the same, without identifiable labels or packaging. The patients will be requested to record the medication and return the packaging to researchers after treatment. The flow chart (Figure 1) shows the simplified procedure of group allocation, interventions, and outcomes assessment.

Termination of therapy (nintedanib or placebo)

(1) The planned end of treatment (till one month after the last cycle of BEP chemotherapy);
(2) Termination of treatment due to severe adverse effects (AEs);
(3) Withdrawal of written informed consent.

Management in case of patients who could not complete pulmonary function test

In patients who may not be able to receive pulmonary function tests due to bleomycin-induced acute lung injury, arterial blood gases (ABGs) and high-resolution pulmonary CT will be performed to indirectly examine the pulmonary function. Meanwhile, it is also an adverse event that will be recorded in this trial. Pulmonary function tests will be performed after recovery or until those patients can complete this examination. For patients whose general condition has deteriorated due to MOGCT progression, if such patients had been enrolled in this trial, they would have recorded baseline and dynamic pulmonary function tests until they could not finish the pulmonary test. If they have not already finished the complete treatment for MOGCT and cannot continuously receive bleomycin-based chemotherapy, these adverse events will be recorded as positive events during the trial to evaluate the safety of nintedanib.

Follow-up data collection

The disease-specific data, including tumor stage, pathological subtypes of MOGCTs, tumor markers' value, surgical options, and chemotherapy response, etc. will further be recorded in detail. The patients need to routinely follow up in outpatient before each chemotherapy cycle. The frequency of outpatient follow-up after finishing chemotherapy is set as follows: every three months within three years after treatment, and every 6 months between 3 and 5 years after treatment. Additional follow-up will be provided in case of special conditions, such as severe chemotherapy side effects, or tumor recurrence. A telephone follow-up will be a supplementary option for those

For peer review only - http://bmjopen.bmj.com/site/about/guidelines.xhtml

patients who follow up in local hospitals or miss the appointment day. Importantly, the patients' smoking status, whether they receive oxygen therapy, and/or chest radiation before or during the treatment will be recorded in detail due to these factors may have a synergized effect with bleomycin.

During each follow-up, the patients will need to report the oncology outcomes, pulmonary function, QoL, menstrual conditions, and fertility outcomes for childbearing patients with the desire for fertility. The interval between bleomycin-containing chemotherapy and the onset of lung injury will be exactly recorded during the trial. The QoL assessment will be based on the general conditions and self-report questionaries listed as follows.

The privacy of patients will be rigorously protected throughout the whole study process. Any statistical analysis or publications of data can only be presented in an anonymization format.

Validated questionaries used in the trial

Quality of life will be mainly assessed by the Functional Assessment of Cancer Therapy Scale General (FACT-G) [23]. Other questionaries include the Insomnia Severity Index (ISI) [24], the Physical Activity Questionnaire (IPAQ) [25], Peripheral Neurotoxicity Questionnaire (GOG-NTX) [26]. The FACT-G is a symptomatic scale including 12 items that can evaluate patients' symptoms, chemotherapy adverse effects, sexual and hormonal situations, etc. Similarly, ISI includes 7 items to assess the quality of sleep and the severity of insomnia. IPAG shows validation in measuring overall physical activity and its frequency and intensity. GOG-NTX uses 11 items to describe peripheral nerve disorders, such as numbness, discomfort, arthralgia, deep sensitivity problems, and walking disorders. The detailed description has also been discussed in a previous study [27]. A combination of these questionaries can help demonstrate the life status of MOGCT patients after treatment.

Outcomes

Primary outcomes

The primary outcome of the study is the declined degree of FVC in MOGCT patients who receive BEP chemotherapy revealed by respiratory function test. The secondary outcomes are the decline of other pulmonary function indices (FEV1; FVC pred%, and DLCO), the safety of nintedanib treatment, oncologic and fertility outcomes, and QoL in MOGCTs patients.

For peer review only

The safety assessment of nintedanib is measured by patients' vital signs, auxiliary examinations, and AEs. The auxiliary examinations include blood tests (routine analysis, liver, and kidney function, etc.), urine, and stool examinations. We will record any AE during the treatment and follow-up period. The definition and classification of AEs based on the CTCAE5.0. Treatment will be continued in case of grade 1 or tolerable grade 2 AEs, and temporary termination of treatment will be applied in case of the first occurrence of intolerable grade 2 or grade 3 AEs until recovery to grade 0-1 or grade 2 AEs. However, permanent termination of nintedanib medication will be considered upon the second time of intolerable grade 2 or grade 3 AEs, no matter the previous toxicities or newly identified toxicities.

Sample size calculation

This study adopted a non-inferiority clinical trial design, and the sample size was calculated by using the sample size calculation website (https://sample-size.net). Based on the decline in lung function of patients treated with bleomycin at 1 year in the literature and the improvement in pulmonary function with nintedanib [17, 28-30], we set the decline of FVC in the placebo group as over 8%. Based on the efficacy of nintedanib in the study of pulmonary fibrosis patients [16], we set the decline of FVC in the nintedanib group as 4%. Using a non-inferiority cut-off value of 10%, a one-sided α value of 0.025, and the test power of 0.09, we calculate the sample size of a single group as 53. Considering the group set a 1:1 ratio in two groups and 10% of the dropout cases during the trial, at least 116 patients will need to be included (Figure S1). Moreover, we presume that no more than 10% of the enrolled MOGCT patients may unable to continue to receive bleomycin-based chemotherapy due to the pulmonary fibrosis, we will recruit additional 10% patients (12 patients) to better conduct this trial. Therefore, we plan to include at least 128 patients in this trial.

Patient and public involvement

We established a panel of 8 patients (in case of adult patients) or their guardians (children or adolescents' patients) was established for this trial. They helped to modify the study design and improve the final study protocol during online focus group meetings. Particularly, they proposed the recommendations on patient-related outcomes including the minimally important clinical

For peer review only

differences, methods of recruitment and the design of data collection. They will be actively involved throughout all critical stages of the trial through regular parent panel meetings. The results of the study will inform the participants or their guardians in case of children/adolescent's participants. Moreover, the additional burden of the intervention in this clinical trial will be fully covered by the hospital. However, the charge due to standard treatment of MOGCTs should be paid by the patients themselves.

Data management and statistical analysis

We will use an electronic case report form (eCRF) to collect data, and an electronic database will be subsequently generated. All the investigators need to be authorized to access the data and will be required to maintain data confidentiality. A periodic indication to update outcomes' information during the follow-up will also be set up. After completion of the study, data will be stored for at least 20 years at a central data drive at the Peking Union Medical College Hospital. The datasets used and/or analyzed during the current study can be obtained from the principal investigator upon reasonable request. Moreover, a written signed data-sharing agreement will be required to get data access.

The variables will be described according to the distributions, namely, mean ± standard deviation (range) for normally distributed continuous variables, otherwise they are described as medians and interquartile ranges. Counts (percentages) will be used to present discrete variables. Methods for comparisons of variables between two groups will also be determined according to the data types. For oncology outcomes, including recurrence-free survival, overall survival, and disease-specific survival, we will use the Kaplan–Meier method (log-rank test). The univariate analysis will be performed to screen factors that should be subjected to multivariate analysis, and we set the cut-off *P*-values as 0.10 in univariate analysis. The Cox regression model or logistic regression model will be conducted in multivariate analysis. SPSS (version 20.0; SPSS Inc., Chicago, IL), GraphPad Prism (version 8.0; GraphPad Software Inc., La Jolla, CA), or STATA (SE v12, StataCorp, College Station, TX, USA) software will be applied to perform the statistical analysis when appropriate. We set the statistical significance cut-off as 0.05 (two-tailed *P*-value).

HARMS

For peer review only

The safety of nintedanib in treating idiopathic pulmonary fibrosis has been confirmed in previous clinical trials [16]. The AEs are usually mild and tolerable. The most common AEs of nintedanib therapy are as follows (incidence ≥ 5%): diarrhea, anorexia, sickness, vomiting, abdominal pain, headache, elevated liver enzyme, weight loss, or hypertension. Other rare AEs reported in previous research include hemorrhage, gastrointestinal perforation, elevated bilirubin levels, and cardiac toxicity. However, currently, there has been limited data on the safety of nintedanib in MOGCT patients receiving bleomycin-based chemotherapy. Therefore, unpredicted AEs may be found during this trial, and all the AEs observed during the study period will be treated according the clinical practice. Especially, for MOGCT patients receiving bleomycin-based chemotherapy, the benefit of prevention of bleomycin-induced pulmonary toxicities would be greater than the risk and the harms of nintedanib itself.

ETHICS AND DISSEMINATION

The Ethics Committee of Peking Union Medical College Hospital has approved the study (I-23PJ400). Written informed consent will be obtained from all participants/guardians. Study results will be submitted to peer-reviewed medical journals for publication and presented at academic conferences. The study will be conducted according to the principles of the Declaration of Helsinki.

CURRENT STATUS

Participants' enrollment is planned to start on Oct. 1st, 2023, and end on April 1st, 2026.

DISCUSSION

At present, bleomycin-induced pulmonary fibrosis remains a severe chemotherapy AE that significantly impairs the therapeutic efficacy and quality of life in MOGCT patients. It was estimated that 8% of patients who received bleomycin may develop varied degrees of pulmonary fibrosis, and nearly 2% were lethal [29]. Although some optional chemotherapy regimens were proposed to decrease chemo toxicities in MOGCT patients, bleomycin is a crucial and irreplaceable regimen for chemotherapy in MOGCTs patients [31, 32]. Nonetheless, the role of bleomycin is more important for recurrent MOGCTs due to dissatisfactory responses to other regimens [6, 12].

For peer review only

Therefore, how to alleviate pulmonary fibrosis during bleomycin-containing chemotherapy in MOGCTs has always raised great concerns.

The metabolism of bleomycin in the lung is poor because of the low activity of detoxifying enzymes and bleomycin hydrolase [8]. Hence, the accumulation of bleomycin in the lung, impairs the vascular system, resulting in the aggregation of inflammatory cells and fibroblasts into the lung tissue, which eventually leads to pulmonary fibrosis [33]. Nonetheless, the exact mechanisms of pulmonary fibrosis development remain unclear, and the time between bleomycin medication and pulmonary fibrosis varies greatly among different patients [34]. Routine prophylactic administration of anti-fibrosis drugs in patients receiving bleomycin may be practical due to the availability for early identification of high-risk patients. In several clinical trials of nintedanib in treating progressive fibrosing interstitial lung diseases or idiopathic pulmonary fibrosis, nintedanib showed a significantly lower reduction in pulmonary function [18, 35]. Furthermore, nintedanib has been showing promising therapeutic efficacy in treating fibrosis caused by bleomycin-containing chemotherapy and potential additional benefits in combination with common chemotherapy regimens in cancer patients [18-20]. Besides, these studies showed relatively minor AEs of nintedanib, revealing the safety of medication in targeted treating patients [35].

However, currently, there is limited evidence of nintedanib for the prevention or treatment of bleomycin-induced pulmonary fibrosis in MOGCT patients. There has been only limited low-grade evidence originating from case reports. Our prospective, double-blinded, randomized-controlled trial aims to provide high-quality on this issue, which will be helpful in the management of the severe AEs caused by bleomycin-containing chemotherapy in this population. Nevertheless, one major limitation of this study is the single-center setting, further multi-center prospective trial should be conducted.

Acknowledgments

We want to thank patient advisers and all the patients enrolled in this study.

Authors' contributions

Jiaxin Yang is the principal investigator of this study who conceives the study design and modifies the manuscript. Sijian Li wrote the manuscript; Tianyu Zhang, Xinyue Zhang, Min Yin, and

For peer review only

Rundong Zhang completed the establishment of the eCRF, participated in the literature review and trial registration; Jie Yang participated in the study design and designed the statistical analysis methods. Sijian Li, Tianyu Zhang, Xinyue Zhang, Min Yin, and Rundong Zhang will participate in patient recruitment, data collection, and statistical analysis. All authors read and approved the manuscript.

Funding

This study was supported by National High-Level Hospital Clinical Research Funding (2022-PUMCH-B-083) and Chinese Academy of Medical Sciences (CAMS) Innovation Fund for Medical Sciences (2022-I2M-C&T-B-023).

Disclaimer

The funding agency had no role in the design, and will not have any role during execution of the trial, data analyses, interpretation of the data, or decision to submit results.

Competing interest

The authors declare no conflict of interest.

Consent to participate and publication

Informed consent to participate and publication will be obtained for each enrolled patient.

Figure

Figure 1. The flow chart demonstrates the simplified process of group allocation, interventions, and outcomes assessment.

Supplementary files.

Table S1. The SPIRIT checklist in this study.

Figure S1. The sample size calculation process in this study.

References:

For peer review only

1. Park M, Lim J, Lee JA, Park HJ, Park BK, Lim MC, et al. Incidence and outcomes of malignant ovarian germ cell tumors in Korea, 1999-2017. Gynecol Oncol. 2021;163(1):79-84. doi: 10.1016/j.ygyno.2021.07.037 PMID: 34392955

2. Smith HO, Berwick M, Verschraegen CF, Wiggins C, Lansing L, Muller CY, et al. Incidence and survival rates for female malignant germ cell tumors. Obstet Gynecol. 2006;107(5):1075-1085. doi: 10.1097/01.AOG.0000216004.22588.ce PMID: 16648414

3. Torre LA, Trabert B, DeSantis CE, Miller KD, Samimi G, Runowicz CD, et al. Ovarian cancer statistics, 2018. CA Cancer J Clin. 2018;68(4):284-296. doi: 10.3322/caac.21456 PMID: 29809280

4. Euscher ED. Germ Cell Tumors of the Female Genital Tract. Surg Pathol Clin. 2019;12(2):621-649. doi: 10.1016/j.path.2019.01.005 PMID: 31097117

5. Ray-Coquard I, Morice P, Lorusso D, Prat J, Oaknin A, Pautier P, et al. Non-epithelial ovarian cancer: ESMO Clinical Practice Guidelines for diagnosis, treatment and follow-up. Ann Oncol. 2018;29(Suppl 4):iv1-iv18. doi: 10.1093/annonc/mdy001 PMID: 29697741

6. Bajorin DF, Bosl GJ. Bleomycin in germ cell tumor therapy: not all regimens are created equal. J Clin Oncol. 1997;15(5):1717-1719. doi: 10.1200/jco.1997.15.5.1717 PMID: 9164177

7. Simone CG, Markham MJ, Dizon DS. Chemotherapy in ovarian germ cell tumors: A systematic review. Gynecol Oncol. 2016;141(3):602-607. doi: 10.1016/j.ygyno.2016.02.007 PMID: 26873864

8. Fyfe AJ, McKay P. Toxicities associated with bleomycin. J R Coll Physicians Edinb. 2010;40(3):213-215. doi: 10.4997/jrcpe.2010.306 PMID: 21127762

9. Allawzi A, Elajaili H, Redente EF, Nozik-Grayck E. Oxidative Toxicology of Bleomycin:

For peer review only

Role of the Extracellular Redox Environment. Curr Opin Toxicol. 2019;13:68-73. doi: 10.1016/j.cotox.2018.08.001 PMID: 31289762

10. Mangili G, Sigismondi C, Gadducci A, Cormio G, Scollo P, Tateo S, et al. Outcome and risk factors for recurrence in malignant ovarian germ cell tumors: a MITO-9 retrospective study. Int J Gynecol Cancer. 2011;21(8):1414-1421. doi: 10.1097/IGC.0b013e3182236582 PMID: 21795985

11. Reddy Ammakkanavar N, Matei D, Abonour R, Einhorn LH. High-dose chemotherapy for recurrent ovarian germ cell tumors. J Clin Oncol. 2015;33(2):226-227. doi: 10.1200/jco.2014.59.4325 PMID: 25452440

12. De Giorgi U, Casadei C, Bergamini A, Attademo L, Cormio G, Lorusso D, et al. Therapeutic Challenges for Cisplatin-Resistant Ovarian Germ Cell Tumors. Cancers (Basel). 2019;11(10). doi: 10.3390/cancers11101584 PMID: 31627378

13. Ortiz LA, Lasky J, Hamilton RF, Jr., Holian A, Hoyle GW, Banks W, et al. Expression of TNF and the necessity of TNF receptors in bleomycin-induced lung injury in mice. Exp Lung Res. 1998;24(6):721-743. doi: 10.3109/01902149809099592 PMID: 9839161

14. Arif M, Basu A, Wolf KM, Park JK, Pommerolle L, Behee M, et al. An Integrative Multiomics Framework for Identification of Therapeutic Targets in Pulmonary Fibrosis. Adv Sci (Weinh). 2023:e2207454. doi: 10.1002/advs.202207454 PMID: 37038090

15. Hilberg F, Roth GJ, Krssak M, Kautschitsch S, Sommergruber W, Tontsch-Grunt U, et al. BIBF 1120: triple angiokinase inhibitor with sustained receptor blockade and good antitumor efficacy. Cancer Res. 2008;68(12):4774-4782. doi: 10.1158/0008-5472.Can-07-6307 PMID: 18559524

For peer review only

16. Flaherty KR, Wells AU, Cottin V, Devaraj A, Walsh SLF, Inoue Y, et al. Nintedanib in Progressive Fibrosing Interstitial Lung Diseases. N Engl J Med. 2019;381(18):1718-1727. doi: 10.1056/NEJMoa1908681 PMID: 31566307

17. Richeldi L, du Bois RM, Raghu G, Azuma A, Brown KK, Costabel U, et al. Efficacy and safety of nintedanib in idiopathic pulmonary fibrosis. N Engl J Med. 2014;370(22):2071-2082. doi: 10.1056/NEJMoa1402584 PMID: 24836310

18. Gundogan BD, Taskinlar S, Arikoglu T, Balci Y, Citak EC. Bleomycin-induced Pneumonitis in a Child Treated With Nintedanib: Report of the First Case in a Childhood. J Pediatr Hematol Oncol. 2022;44(2):e500-e502. doi: 10.1097/mph.0000000000002266 PMID: 35200223

19. Kai Y, Matsuda M, Fukuoka A, Hontsu S, Yamauchi M, Yoshikawa M, et al. Remarkable response of non-small cell lung cancer to nintedanib treatment in a patient with idiopathic pulmonary fibrosis. Thorac Cancer. 2021;12(9):1457-1460. doi: 10.1111/1759-7714.13935 PMID: 33742758

20. Kato R, Haratani K, Hayashi H, Sakai K, Sakai H, Kawakami H, et al. Nintedanib promotes antitumour immunity and shows antitumour activity in combination with PD-1 blockade in mice: potential role of cancer-associated fibroblasts. Br J Cancer. 2021;124(5):914-924. doi: 10.1038/s41416-020-01201-z PMID: 33299131

21. Cheng CW, Wu TX, Shang HC, Li YP, Altman DG, Moher D, et al. CONSORT Extension for Chinese Herbal Medicine Formulas 2017: Recommendations, Explanation, and Elaboration. Ann Intern Med. 2017;167(2):112-121. doi: 10.7326/m16-2977 PMID: 28654980

22. Chan AW, Tetzlaff JM, Altman DG, Laupacis A, Gøtzsche PC, Krleža-Jerić K, et al. SPIRIT 2013 statement: defining standard protocol items for clinical trials. Ann Intern Med.

For peer review only

436 2013;158(3):200-207. doi: 10.7326/0003-4819-158-3-201302050-00583 PMID: 23295957

437 23. King MT, Stockler MR, Cella DF, Osoba D, Eton DT, Thompson J, et al. Meta-analysis
438 provides evidence-based effect sizes for a cancer-specific quality-of-life questionnaire, the
439 FACT-G. J Clin Epidemiol. 2010;63(3):270-281. doi: 10.1016/j.jclinepi.2009.05.001 PMID:
440 19716264

441 24. Morin CM, Belleville G, Bélanger L, Ivers H. The Insomnia Severity Index: psychometric
442 indicators to detect insomnia cases and evaluate treatment response. Sleep. 2011;34(5):601-
443 608. doi: 10.1093/sleep/34.5.601 PMID: 21532953

444 25. Keating XD, Zhou K, Liu X, Hodges M, Liu J, Guan J, et al. Reliability and Concurrent
445 Validity of Global Physical Activity Questionnaire (GPAQ): A Systematic Review. Int J Environ
446 Res Public Health. 2019;16(21). doi: 10.3390/ijerph16214128 PMID: 31717742

447 26. Cheng HL, Molassiotis A. Longitudinal validation and comparison of the Chinese version
448 of the European Organization for Research and Treatment of Cancer Quality of Life-
449 Chemotherapy-Induced Peripheral Neuropathy Questionnaire (EORTC QLQ-CIPN20) and the
450 Functional Assessment of Cancer-Gynecologic Oncology Group-Neurotoxicity subscale
451 (FACT/GOG-Ntx). Asia Pac J Clin Oncol. 2019;15(1):56-62. doi: 10.1111/ajco.13000 PMID:
452 29873180

453 27. Gernier F, Ahmed-Lecheheb D, Pautier P, Floquet A, Nadeau C, Frank S, et al. "Chronic
454 fatigue, quality of life and long-term side-effects of chemotherapy in patients treated for non-
455 epithelial ovarian cancer: national case-control protocol study of the GINECO-Vivrovaire rare
456 tumors INCa French network for rare malignant ovarian tumors". BMC Cancer.
457 2021;21(1):1147. doi: 10.1186/s12885-021-08864-8 PMID: 34702204

For peer review only

28. Wollin L, Distler JHW, Redente EF, Riches DWH, Stowasser S, Schlenker-Herceg R, et al. Potential of nintedanib in treatment of progressive fibrosing interstitial lung diseases. Eur Respir J. 2019;54(3). doi: 10.1183/13993003.00161-2019 PMID: 31285305

29. Delanoy N, Pécuchet N, Fabre E, Combe P, Juvin K, Pujade-Lauraine E, et al. Bleomycin-Induced Pneumonitis in the Treatment of Ovarian Sex Cord-Stromal Tumors: A Systematic Review and Meta-analysis. Int J Gynecol Cancer. 2015;25(9):1593-1598. doi: 10.1097/igc.0000000000000530 PMID: 26308607

30. Roncolato FT, Chatfield M, Houghton B, Toner G, Stockler M, Thomson D, et al. The effect of pulmonary function testing on bleomycin dosing in germ cell tumours. Intern Med J. 2016;46(8):893-898. doi: 10.1111/imj.13158 PMID: 27305276

31. Chu R, Liu P, Chen J, Cheng X, Li K, Che Y, et al. Fertility and prognosis assessment between bleomycin/etoposide/cisplatin and paclitaxel/carboplatin chemotherapy regimens in the conservative treatment of malignant ovarian germ cell tumors: a multicenter and retrospective study. J Gynecol Oncol. 2023;34(2):e12. doi: 10.3802/jgo.2023.34.e12 PMID: 36890292

32. Satoh T, Aoki Y, Kasamatsu T, Ochiai K, Takano M, Watanabe Y, et al. Administration of standard-dose BEP regimen (bleomycin+etoposide+cisplatin) is essential for treatment of ovarian yolk sac tumour. Eur J Cancer. 2015;51(3):340-351. doi: 10.1016/j.ejca.2014.12.004 PMID: 25559616

33. Huang TT, Hudson MM, Stokes DC, Krasin MJ, Spunt SL, Ness KK. Pulmonary outcomes in survivors of childhood cancer: a systematic review. Chest. 2011;140(4):881-901. doi: 10.1378/chest.10-2133 PMID: 21415131

For peer review only

34. Sleijfer S. Bleomycin-induced pneumonitis. Chest. 2001;120(2):617-624. doi: 10.1378/chest.120.2.617 PMID: 11502668

35. Bendstrup E, Wuyts W, Alfaro T, Chaudhuri N, Cornelissen R, Kreuter M, et al. Nintedanib in Idiopathic Pulmonary Fibrosis: Practical Management Recommendations for Potential Adverse Events. Respiration. 2019;97(2):173-184. doi: 10.1159/000495046 PMID: 30544129

For peer review only

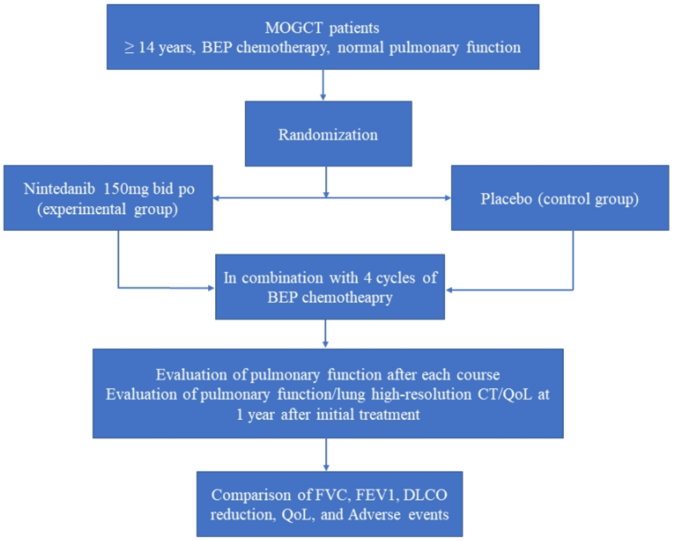

338x190mm (96 x 96 DPI)

The standard normal deviate for α = Z_α = 1.9600
The standard normal deviate for β = Z_β = 0.8416
Pooled proportion = P = $(q_1 * P_1) + (q_0 * P_0)$ = 0.1450
$A = Z_\alpha \sqrt{P(1-P)(1/q_1 + 1/q_0)}$ = 1.3802
$B = Z_\beta \sqrt{P_1(1-P_1)(1/q_1) + P_0(1-P_0)(1/q_0)}$ = 0.5657
$C = (P_1 - P_0)^2$ = 0.0441
Total group size = N = $(A+B)^2/C$ = 86
Continuity correction (added to N for Group 0) = CC = $1/(q_1 * |P_1 - P_0|)$ = 10

Sample size (with continuity correction)

	N	Outcome+	Outcome-
Group 1:	53	2	51
Group 0:	53	13	40
Total:	106	15	91

For peer review only

STANDARD PROTOCOL ITEMS: RECOMMENDATIONS FOR INTERVENTIONAL TRIALS

SPIRIT 2013 Checklist: Recommended items to address in a clinical trial protocol and related documents*

Section/item	ItemNo	Description	Lines
**Administrative information**			
Title	1	Descriptive title identifying the study design, population, interventions, and, if applicable, trial acronym	1-3
Trial registration	2a	Trial identifier and registry name. If not yet registered, name of intended registry	36-37
	2b	All items from the World Health Organization Trial Registration Data Set	36-37
Protocol version	3	Date and version identifier	36-37
Funding	4	Sources and types of financial, material, and other support	319-321
Roles and responsibilities	5a	Names, affiliations, and roles of protocol contributors	323-325
	5b	Name and contact information for the trial sponsor	319-321
	5c	Role of study sponsor and funders, if any, in study design; collection, management, analysis, and interpretation of data; writing of the report; and the decision to submit the report for publication, including whether they will have ultimate authority over any of these activities	323-325
	5d	Composition, roles, and responsibilities of the coordinating centre, steering committee, endpoint adjudication committee, data management team, and other individuals or groups overseeing the trial, if applicable (see Item 21a for data monitoring committee)	323-325
**Introduction**			

For peer review only

Background and rationale	6a	Description of research question and justification for undertaking the trial, including summary of relevant studies (published and unpublished) examining benefits and harms for each intervention	50-88
	6b	Explanation for choice of comparators	50-88
Objectives	7	Specific objectives or hypotheses	91-96
Trial design	8	Description of trial design including type of trial (eg, parallel group, crossover, factorial, single group), allocation ratio, and framework (eg, superiority, equivalence, noninferiority, exploratory)	100-101
**Methods: Participants, interventions, and outcomes**			
Study setting	9	Description of study settings (eg, community clinic, academic hospital) and list of countries where data will be collected. Reference to where list of study sites can be obtained	101-103
Eligibility criteria	10	Inclusion and exclusion criteria for participants. If applicable, eligibility criteria for study centres and individuals who will perform the interventions (eg, surgeons, psychotherapists)	118-136
Interventions	11a	Interventions for each group with sufficient detail to allow replication, including how and when they will be administered	108-115; 154-163
	11b	Criteria for discontinuing or modifying allocated interventions for a given trial participant (eg, drug dose change in response to harms, participant request, or improving/worsening disease)	168-184
	11c	Strategies to improve adherence to intervention protocols, and any procedures for monitoring adherence (eg, drug tablet return, laboratory tests)	162-163
	11d	Relevant concomitant care and interventions that are permitted or prohibited during the trial	154-157
Outcomes	12	Primary, secondary, and other outcomes, including the specific measurement variable (eg, systolic blood pressure), analysis metric (eg, change from baseline, final value, time to event), method of aggregation (eg, median, proportion), and time point for each outcome. Explanation of the clinical relevance of chosen efficacy and harm outcomes is strongly recommended	220-232

For peer review only - http://bmjopen.bmj.com/site/about/guidelines.xhtml

Participant timeline	13	Time schedule of enrolment, interventions (including any run-ins and washouts), assessments, and visits for participants. A schematic diagram is highly recommended (see Figure)	186-202; 274
Sample size	14	Estimated number of participants needed to achieve study objectives and how it was determined, including clinical and statistical assumptions supporting any sample size calculations	235-243
Recruitment	15	Strategies for achieving adequate participant enrolment to reach target sample size	140-144
**Methods: Assignment of interventions (for controlled trials)**			
Allocation:			
Sequence generation	16a	Method of generating the allocation sequence (eg, computer-generated random numbers), and list of any factors for stratification. To reduce predictability of a random sequence, details of any planned restriction (eg, blocking) should be provided in a separate document that is unavailable to those who enrol participants or assign interventions	151-154
Allocation concealment mechanism	16b	Mechanism of implementing the allocation sequence (eg, central telephone; sequentially numbered, opaque, sealed envelopes), describing any steps to conceal the sequence until interventions are assigned	151-154
Implementation	16c	Who will generate the allocation sequence, who will enrol participants, and who will assign participants to interventions	152-153
Blinding (masking)	17a	Who will be blinded after assignment to interventions (eg, trial participants, care providers, outcome assessors, data analysts), and how	152-153
	17b	If blinded, circumstances under which unblinding is permissible, and procedure for revealing a participant's allocated intervention during the trial	151-154
**Methods: Data collection, management, and analysis**			
Data collection methods	18a	Plans for assessment and collection of outcome, baseline, and other trial data, including any related processes to promote data quality (eg, duplicate measurements, training of assessors) and a description of study instruments (eg, questionnaires, laboratory tests) along with their reliability and validity, if known. Reference to where data collection forms can be found, if not in the protocol	187-201

	18b	Plans to promote participant retention and complete follow-up, including list of any outcome data to be collected for participants who discontinue or deviate from intervention protocols	187-201
Data management	19	Plans for data entry, coding, security, and storage, including any related processes to promote data quality (eg, double data entry; range checks for data values). Reference to where details of data management procedures can be found, if not in the protocol	246-253
Statistical methods	20a	Statistical methods for analysing primary and secondary outcomes. Reference to where other details of the statistical analysis plan can be found, if not in the protocol	254-265
	20b	Methods for any additional analyses (eg, subgroup and adjusted analyses)	254-265
	20c	Definition of analysis population relating to protocol non-adherence (eg, as randomised analysis), and any statistical methods to handle missing data (eg, multiple imputation)	174-184
**Methods: Monitoring**			
Data monitoring	21a	Composition of data monitoring committee (DMC); summary of its role and reporting structure; statement of whether it is independent from the sponsor and competing interests; and reference to where further details about its charter can be found, if not in the protocol. Alternatively, an explanation of why a DMC is not needed	187-201
	21b	Description of any interim analyses and stopping guidelines, including who will have access to these interim results and make the final decision to terminate the trial	187-201
Harms	22	Plans for collecting, assessing, reporting, and managing solicited and spontaneously reported adverse events and other unintended effects of trial interventions or trial conduct	224-232
Auditing	23	Frequency and procedures for auditing trial conduct, if any, and whether the process will be independent from investigators and the sponsor	269-271
**Ethics and dissemination**			

Research ethics approval	24	Plans for seeking research ethics committee/institutional review board (REC/IRB) approval	32-33; 268-269
Protocol amendments	25	Plans for communicating important protocol modifications (eg, changes to eligibility criteria, outcomes, analyses) to relevant parties (eg, investigators, REC/IRBs, trial participants, trial registries, journals, regulators)	100-103
Consent or assent	26a	Who will obtain informed consent or assent from potential trial participants or authorised surrogates, and how (see Item 32)	269
	26b	Additional consent provisions for collection and use of participant data and biological specimens in ancillary studies, if applicable	269
Confidentiality	27	How personal information about potential and enrolled participants will be collected, shared, and maintained in order to protect confidentiality before, during, and after the trial	246-253
Declaration of interests	28	Financial and other competing interests for principal investigators for the overall trial and each study site	328
Access to data	29	Statement of who will have access to the final trial dataset, and disclosure of contractual agreements that limit such access for investigators	249-253
Ancillary and post-trial care	30	Provisions, if any, for ancillary and post-trial care, and for compensation to those who suffer harm from trial participation	268-271
Dissemination policy	31a	Plans for investigators and sponsor to communicate trial results to participants, healthcare professionals, the public, and other relevant groups (eg, via publication, reporting in results databases, or other data sharing arrangements), including any publication restrictions	268-271
	31b	Authorship eligibility guidelines and any intended use of professional writers	268-271
	31c	Plans, if any, for granting public access to the full protocol, participant-level dataset, and statistical code	252-253
**Appendices**			
Informed consent materials	32	Model consent form and other related documentation given to participants and authorised surrogates	331

For peer review only - http://bmjopen.bmj.com/site/about/guidelines.xhtml

Biological specimens	33	Plans for collection, laboratory evaluation, and storage of biological specimens for genetic or molecular analysis in the current trial and for future use in ancillary studies, if applicable	NA

*It is strongly recommended that this checklist be read in conjunction with the SPIRIT 2013 Explanation & Elaboration for important clarification on the items. Amendments to the protocol should be tracked and dated. The SPIRIT checklist is copyrighted by the SPIRIT Group under the Creative Commons "Attribution-NonCommercial-NoDerivs 3.0 Unported" license.

For peer review only
